# Antisense targeting of FOXP3+ Tregs to boost anti-tumor immunity

**DOI:** 10.3389/fimmu.2024.1426657

**Published:** 2024-08-21

**Authors:** Tatiana Akimova, Liqing Wang, Zhanna Bartosh, Lanette M. Christensen, Evgeniy Eruslanov, Sunil Singhal, Veenu Aishwarya, Wayne W. Hancock

**Affiliations:** ^1^ Division of Transplant Immunology, Department of Pathology and Laboratory Medicine, Children’s Hospital of Philadelphia and Perelman School of Medicine at the University of Pennsylvania, Philadelphia, PA, United States; ^2^ AUM Biotech, LLC., Philadelphia, PA, United States; ^3^ Division of Thoracic Surgery, Department of Surgery, Perelman School of Medicine at the University of Pennsylvania, Philadelphia, PA, United States

**Keywords:** ASO, antisense oligonucleotides, FOXP3, Tregs, cancer, immunotherapy

## Abstract

Our goal is to improve the outcomes of cancer immunotherapy by targeting FOXP3+ T-regulatory (Treg) cells with a next generation of antisense oligonucleotides (ASO), termed FOXP3 AUMsilence ASO. We performed *in vitro* experiments with human healthy donor PBMC and clinical samples from patients with lung cancer, mesothelioma and melanoma, and tested our approach *in vivo* using ASO FOXP3 in syngeneic murine cancer models and in humanized mice. ASO FOXP3 had no effects on cell viability or cell division, did not affect expression of other FOXP members, but decreased expression of FOXP3 mRNA in PBMC by 54.9% and in cancer samples by 64.7%, with corresponding 41.0% (PBMC) and 60.0% (cancer) decreases of Treg numbers (all p<0.0001). Hence, intratumoral Treg were more sensitive to the effects of ASO FOXP3 than peripheral blood Tregs. Isolated human Treg, incubated with ASO FOXP3 for 3.5 hours, had significantly impaired suppressive function (66.4%) versus Scramble control. In murine studies, we observed a significant inhibition of tumor growth, while 13.6% (MC38) to 22% (TC1) of tumors were completely resorbed, in conjunction with ~50% decrease of Foxp3 mRNA by qPCR and decreased numbers of intratumoral Tregs. In addition, there were no changes in FOXP3 mRNA expression or in the numbers of Tregs in draining lymph nodes and in spleens of tumor bearing mice, confirming that intratumoral Treg had enhanced sensitivity to ASO FOXP3 *in vivo* compared to other Treg populations. ASO FOXP3 Treg targeting *in vivo* and *in vitro* was accompanied by significant downregulation of multiple exhaustion markers, and by increased expression of perforin and granzyme-B by intratumoral T cells. To conclude, we report that targeting the key Treg transcription factor FOXP3, with ASO FOXP3, has a powerful anti-tumoral effect and enhances T cell response *in vitro* and *in vivo*.

## 1 Introduction

Regulatory FOXP3+ T cells (Tregs) are key mediators of tolerance, mitigating excessive and harmful immune responses to maintain immune homeostasis (1, 2). However, Tregs can prevent effective anti-tumor immune responses (3–8) by suppressing T cells and antigen-presenting cells, releasing immunosuppressive molecules such as TGF-β, IL-10 and IL-35, activating apoptosis, promoting exhaustion of intratumoral T cells, and other actions (9). As a result, targeting of intratumoral Tregs is now seen as a critical aim of anti-cancer immunotherapy (7, 10, 11). To date, various methods of targeting Tregs have been tested, including use of anti-CD4, anti-CD25 or anti-CTLA-4 monoclonal antibodies (12–14), cyclophosphamide (15, 16), diphtheria toxin conjugated to IL-2 (17), and small molecule inhibitors to disrupt signals promoting Treg function (7, 18–20). Unfortunately, these approaches have demonstrated limited success due to co-targeting of CD4+ and/or CD25+ conventional T cells and other immune cells (21, 22), or rebound of Treg numbers (23–25). Another problem of systemic Treg depletion is uncontrolled activation of immune system with an onset of autoimmunity (26–29), since extratumoral Treg cells are central to immune homeostasis.

It is thus imperative to develop selective methods for targeting the function of Tregs in tumors while leaving systemic Tregs unaffected. This is a challenging task due to two problems: an absence of unique Treg cell surface markers to target them versus other T cells with antibodies, and an absence of any unique markers of intratumoral Tregs in comparison with conventional Treg cells (30). However, Tregs express a key transcription factor and master regulator, FOXP3, that controls the majority of Treg-specific genes required for their cellular identity, development and function (1, 2, 8), and whose sequence is 91% conserved between mice and humans (31). Therefore, targeting FOXP3 using RNA silencing could be a promising approach to downregulate FOXP3 to alleviate Treg-mediated immunosuppression. Our current study builds on our previous work showing remarkably high levels of FOXP3 protein and FOXP3 mRNA in intratumoral Tregs vs. conventional Tregs (30). We propose this difference leads to preferential sensitivity of intratumoral vs. other Tregs. To implement this approach, we utilized a new, highly improved type of antisense oligonucleotide (ASO) directed toward FOXP3 that uses a 2’-deoxy-2’-fluoro-beta-D-arabinose sugar modification (FANA) of single stranded ASO with a phosphothioate backbone, comprising a DNA segment flanked by FANA segments. FOXP3 AUMsilence ASO do not require delivery agents *in vitro* or *in vivo* and are appropriate for use in primary cells (32–38). This type of ASO belongs to the 3^rd^ generation of ASOs with enhanced stability and potency (39–42). Unlike other 2’ modified ASO analogs, AUMsilence FANA ASO is considered a DNA mimic, it forms ASO: RNA hybrids that mimic the structure of the native DNA: RNA hybrid and supports RNase H activity (39, 43, 44).

## 2 Materials and methods

### 2.1 Donors

Healthy donor peripheral blood mononuclear cells (PBMCs) were obtained through the University of Pennsylvania Human Immunology Core. 26 donors were evaluated, mean age 33.9 ± 1.9 years (mean ± SEM), 38.5% males. We also studied three rejected lung transplant samples, the reasons for rejections were profound ischemia, inflammation or combination of both factors. All donors provided informed consent.

### 2.2 Cancer patients

We studied 17 patients with lung cancer, 1 patient with melanoma and 1 patient with mesothelioma, aged 63.1 ± 2.1 years old, 6 males, 7 females and 5 of unknown gender. Tumor samples and tumor-free distant lung samples were received from patients undergoing lung surgery including metastatic melanoma, adenocarcinoma tumors grade T1a, T1b and T2a, large cell neuroendocrine carcinoma T3, squamous cell carcinomas T1a and T2b, typical carcinoid tumor T2a and mesothelioma. None of these patients, except for the subject with melanoma, received previous chemotherapy treatment. Pleural effusion samples were received from 7 patients with stage IV lung adenocarcinoma, all of whom had received previous chemotherapy treatment with pemetrexed, carboplatin and pembrolizumab. The study was approved by local institutional review boards, IRB#813004 and #823659, UPenn.

### 2.3 Human cell isolation and culture

Surgically removed lung tumors from patients were processed within 30 minutes of resection and digested with an enzymatic cocktail for 45–95 minutes with shaking, as described (30) and detailed in **Supplementary Materials**. We isolated PBMCs from blood using standard Ficoll technique, and prepared single cell suspensions from LNs using mechanical dissection as described (45).

### 2.4 Treg isolation, culture and suppression assays

We isolated healthy donor CD4+CD25+Tregs by human Treg isolation kit (Miltenyi Biotec, 130–091-301). For gene expression assays, Treg were stimulated with CD3/28 microbeads, 1.3 beads per cell (**Supplementary Table 1**) and incubated overnight with 1.5 μM of Scramble or ASO FOXP3. For suppression assays, Tregs were incubated with 2.5 μM of ASO Scramble or ASO FOXP3 for 3.5 hours, washed twice and used as described (45). Briefly, we labeled healthy donor PBMC samples with CFSE (5 μM, Invitrogen), stimulated them with CD3 microbeads (1.3 beads/cell, **Supplementary Table 1**) and incubated the cells for 5–6 days with serially diluted pre-treated Tregs at ratios of 1:1 to 1:16 Treg: PBMC. Suppressive function was determined as the area under the curve, as described previously (45). To study the effects of ASO FOXP3 on Treg-depleted samples, we used cells that were depleted of CD4+CD25+ Tregs using Treg isolation kit (Miltenyi Biotec, 130–091-301).

### 2.5 Screening experiments with PBMC for evaluation of ASO FOXP3 toxicity

Healthy donors PBMC were labeled with CFSE and stimulated with CD3 microbeads, 1.3 beads/cell, for 5 days, then evaluated by flow cytometry. Data were calculated as percent of changes over control: 100*[(current ASO result – Scramble result)/Scramble result)].

### 2.6 Post-screening in vitro experiments with ASO

Cells were stimulated with CD3/28 microbeads, 0.3 beads/cell and treated with 1.5 μM of Scramble or ASO FOXP3 for 5 days. We used DMEM with 3% of FBS, and added NH_4_Cl and Arsenic, as described in **Supplementary Materials**. To study exTregs, we labeled healthy donors PBMC with APC-conjugated CD25 antibodies (**Supplementary Table 1**), washed and treated cells as described above. Small aliquots of pre-treated PBMC samples were used to evaluate quality of Treg labeling by co-staining with FOXP3. In the end of experiment, CD4+CD25+FOXP3+ cells represented Tregs (or “pre-existing Tregs”), CD4+CD25+FOXP3- cells represented exTregs, CD4+CD25-FOXP3+ cells represented “*de novo*” Tregs (or CD4+ conventional cells which upregulated FOXP3) and CD4+CD25-FOXP3- cells represented “conventional T cells”.

### 2.7 Mice

For tumor models, we used 6–8 weeks old C57BL/6 mice (both genders) from The Jackson Laboratory, Bar Harbor, ME, USA. TC1 adenocarcinoma cells were provided by Y. Paterson (U. Penn) and MC38 cells, derived from C57BL6 murine colon adenocarcinoma cells, were obtained from NCI/NIH. Cells were grown in RPMI, 10% fetal bovine serum (FBS), 2 mM glutamine, and 5 μg/ml of penicillin and streptomycin. Each mouse was injected subcutaneously on its right flank with 1.2 × 10^6^ TC1 or MC38 cells. At day 7 after tumor inoculation, mice were randomly divided into groups to receive phosphate-buffered saline (PBS), Scramble control or ASO FOXP3 treatment; mice received daily i.p. injections of 50 mg/kg for 14–16 days, depending on tumor growth. Tumor volumes were measured every 2–3 days, and tumor volume was determined by the following formula: (3.14 × long axis × short axis × short axis)/6, as described (46). To combine data of tumor growth from different experiments, data were normalized within each experiment using 0–100 normalization, as to an average of the first day tumor sizes in Scramble group was set up as 0, and the average of the tumor sizes in Scramble group at the last day was set as 100. To study intratumoral events, another set of mice was sacrificed at day 14–15 after tumor inoculation, after a week of treatment. Murine tumors were processed similarly to their human counterparts, as described above. Single cell suspensions from lymph nodes and spleens were prepared as described (45). Mice were studied using the protocols approved by the Institutional Animal Care and Use Committee of the Children’s Hospital of Philadelphia (IAC 22–001047).

Humanized mice PBMC-Hu-NSG, strain #005557, transplanted with human PBMC, were obtained from The Jackson Laboratory and housed in a barrier room in aseptic conditions. Mice were treated with Scramble control or human ASO FOXP3 by i.p. injections 50 mg/kg daily for 4 days, followed by collection of blood, spleens and LNs for qPCR.

RAG1-/- mice, strain #034159, were obtained from The Jackson Laboratory and housed in a barrier room in aseptic conditions. Six mice were injected i.v. with 0.74x10^6^ Thy1.1 CD4+CD25- T cells (strain # 000406, Jax) and with 0.74x10^6^ Thy1.2 CD4+CD25+ Tregs (C57BL/6 mice, Jax), and treated with Scramble control or mouse ASO FOXP3 by i.p. injections 50 mg/kg daily for 7 days, followed by collection of spleens for flow cytometry. CD4+CD25+ Tregs and CD4+CD25- conventional T cells for injections were isolated by mouse Treg isolation kit (Miltenyi Biotec, 130–091-041). Thy1.1+ CD4+FOXP3+ cells represented “*de novo* Tregs”, Thy1.1+CD4+FOXP3- cells represented “conventional Thy1.1 CD4+ T cells”, Thy1.2+CD4+FOXP3+ cells represented “Tregs” and Thy1.2+CD4+FOXP3- cells represented “exTregs”.

### 2.8 Murine cell culture

To evaluate cytokine production, murine samples were stimulated for 4 hours with a cocktail of PMA (phorbol myristate acetate, 3 ng/ml) and ionomycin (1 µM) + Monensin (Biolegend) and evaluated by flow cytometry.

### 2.9 Histology

Lungs, livers, colon and skin samples from TC1 tumor bearing mice, treated with Scramble control or ASO FOXP3 50 mg/kg i.p. daily, were fixed in 10% formalin, and then routinely processed and embedded in paraffin. Histological sections were stained with hematoxylin and eosin (H&E) and were reviewed by a pathologist (W.W.H.).

### 2.10 Flow cytometry

Was performed as following: live/dead fixable reagent, Fc block and monocytes block, superficial staining, then fixation and permeabilization and then staining with antibodies for intranuclear (FOXP3) or intracellular (cytokines, CTLA-4) targets. Details and reagents are described in **Supplementary Materials** and in **Supplementary Table 1**.

### 2.11 RT-qPCR

We isolated RNA with the RNeasy kit (Qiagen), RNAqueous kit or Trizol (both Invitrogen) synthesized cDNA (N8080234, ThermoFisher Scientific), and ran TaqMan gene expression assays with primers from ThermoFisher Scientific, as detailed in **Supplementary methods** and in the **Supplementary Table 1**. We used delta-delta Ct method for calculating relative gene expression. To compare gene expression between two groups of samples (murine experiments), we applied delta-delta Ct method variation as described in (47).

### 2.12 Statistics

All data were tested for normal distribution of variables, and then corresponding parametric or nonparametric tests were used, as indicated in Figure legends. P values of less than 0.05 were considered significant. Figures show mean ± SE. For flow cytometry data statistics and for PCA analysis (detailed in **Supplementary methods**), data were transformed by Z-scoring to make data from different experiments in two tumor models comparable.

## 3 Results

### 3.1 Development and initial screening of 19 human ASO FOXP3 candidates

**Figure 1:**
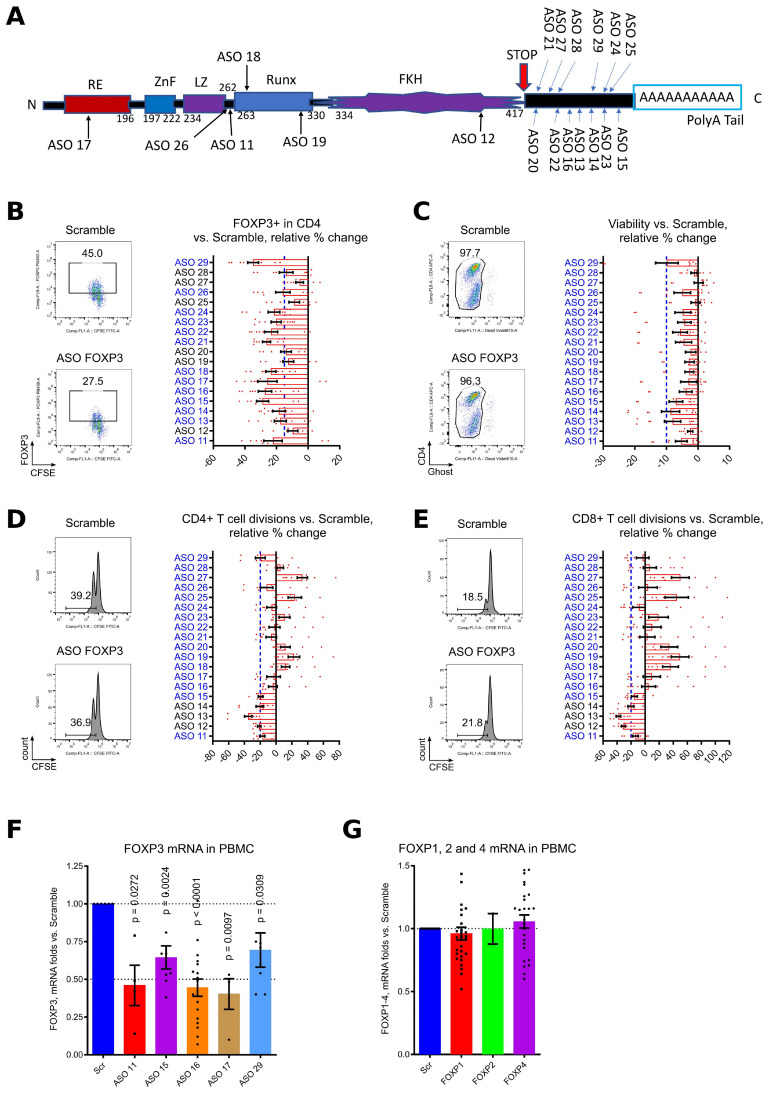
Initial screening of 19 ASO FOXP3 with healthy donors PBMC. **(A)** an alignment of 19 ASO FOXP3 with published human FOXP3 sequence. “RE” – repressor domain, “ZnF” - C2H2 zinc finger region, “LZ” - leucine zipper domain, “Runx” - runt-related transcription factor 1 at CNS2 region, “FKH” - forkhead domain. **(B-E)** PBMC from 7 different donors in 5 experiments were labeled with CFSE and stimulated for 4–5 days with CD3 microbeads 1.3 beads/cell in presence of 1.5 μM Scramble controls of ASOs, and then evaluated by flow cytometry. Flow cytometry data counted as percent of changes over control: 100*((current ASO result – Scramble result)/Scramble result). **(B)** evaluation of FOXP3 expression, cut-off was set up at 15% decrease of FOXP3 vs. Scramble, blue dotted line. The representative staining (on the left) and all data (on the right) are shown. ASOs which passed this screening, are blue. **(C)** evaluation of cellular toxicity by cell viability. Cells were stained for Ghost live/dead fixable reagent. Cut-off was set up at 10%, blue dotted line. The representative staining (on the left) and all data (on the right) are shown. ASOs which passed this screening, are blue. **(D, E)** evaluation of toxicity by inhibition of CD4+ **(D)** and CD8+ **(E)** T cell divisions rates. Cut-off was set up at 20%, blue dotted line. The representative staining with CFSE plots (on the left) and all data (on the right) are shown. ASOs which passed this screening, are blue. ASO 11, 15, 16, 17, 18, 21, 22, 23, 24, 26 and 29 passed all three screening tests. **(F, G)** PBMC from 11 donors in 9 experiments were stimulated with CD3/28 microbeads, 0.3 beads/cell and treated with 1.5μM of Scramble or ASOs for 5 days, then evaluated by qPCR for FOXP3 mRNA expression **(F)** and for mRNA expression of FOXP family genes **(G)**. FOXP2 expression was below the detection limit for the majority of PBMC samples from healthy donors. (**F, G)** one sample T-tests with mean =1, only results with p<0.05 are shown.

We designed 19 FOXP3 AUMsilence ASOs directed against different regions of FOXP3 (**Figure 1A**) (48) with a main focus on its 3’ untranslated region, and performed a series of screening tests using healthy donor PBMC, stimulated with CD3 microbeads in the presence of 1.5 μM of each ASO FOXP3 or Scramble for 4–5 days. The following criteria were applied: a) decrease in the number of FOXP3+ cells greater than or equal to 15% compared to Scramble; b) no more than 10% decrease in cell viability; and c) negligible (20% or less) inhibition of cellular division by CD4+ and CD8+ T cells. 11 out of 19 ASO FOXP3 candidates passed these criteria (**Figures 1B–E**). Next, we confirmed that downregulation of FOXP3 protein was accompanied by downregulation of FOXP3 mRNA expression (**Figure 1F**). Importantly, ASO FOXP3 did not significantly downregulate gene expression of any other members of the FOXP family, confirming that ASO targeting of FOXP3 is highly specific (**Figure 1G**).

### 3.2 Effects of ASOs FOXP3 on human Tregs

**Figure 2:**
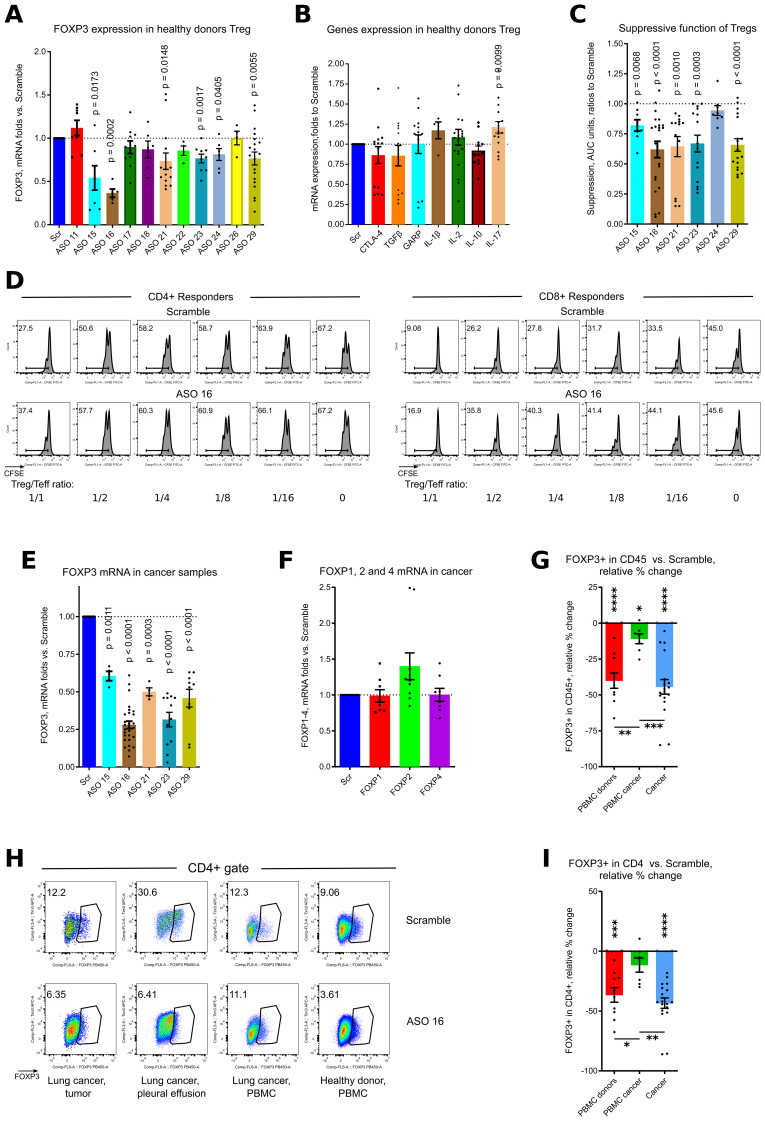
ASO FOXP3 effects on healthy donors Tregs and in cancer samples. **(A)** healthy donor Tregs, isolated from 9 donors in 9 experiments were stimulated with CD3/28 microbeads, 1.3 beads/cell, in presence of 1.5 μM of Scramble or ASOs FOXP3 for overnight, then evaluated by qPCR. More data are shown in **Supplementary Figure 1A**. **(B)** same Tregs as in **(A)** were evaluated for mRNA expression of corresponding genes. **(C, D)**. healthy donor Tregs, isolated from 13 donors in 13 experiments were incubated with 2.5 μM of Scramble of ASO FOXP3 for 3.5 hours, then washed twice and used in 5–6 days suppression assay with autologous or allogeneic responders from 10 donors. **(C)** statistics of suppression assays with the best ASO candidates are shown. **(D)** representative CFSE plots are shown. More data are shown in **Supplementary Figure 1B**. **(E, F)** 3 lung cancer and 1 melanoma tumor samples, 6 pleural effusion samples, 4 lung cancer distant lung and 2 lymph nodes from 11 patients with lung cancer in 7 experiments, were stimulated with CD3/28 microbeads, 0.3 beads/cell and treated with 1.5μM of Scramble or ASOs for 5 days, then evaluated by qPCR. More data are shown in **Supplementary Figure 1C**. **(F)** same samples as in **(E)** were evaluated for mRNA expression of FOXP1, FOXP2 and FOXP4. **(G-I)** multiple cancer, non-cancer and healthy donors PBMC samples were evaluated in five flow cytometry experiments with 2–3 different panels for each experiment. Cells were stimulated with CD3/28 microbeads, 0.3 beads/cell and treated with 1.5μM of Scramble or ASO 16 for 5 days. Samples consisted of: 5 healthy donors PBMC, 3 lung cancer tumor samples and 1 mesothelioma tumor sample, 4 tumor-free lung cancer samples received during a surgery, 3 lung cancer pleural effusion samples, 3 lung draining lymph nodes cancer samples and 4 lung cancer PBMC samples from 10 lung cancer and 1 mesothelioma patients. **(G, I)** statistics of FOXP3 expression in viable CD45+ **(G)** and in CD4+ **(I)** cells. Relative % change calculated as 100*((ASO 16 result – Scramble result)/Scramble result). For this calculation, we used the averaged values for a single sample evaluated in different flow cytometry panels, and comparison was calculated versus two (more often) or one independent Scramble controls. **(H)** Representative flow cytometry plots of FOXP3+ expression in CD4+ cells. **(A-C, E, F)** one sample T-tests with mean =1, only results with p<0.05 are shown. **(G-I)** for PBMC donors and PBMC cancer - one sample T-tests with mean = 0; cancer – one sample Wilcoxon rank test with median =0, only results with p<0.05 are shown using asterisks above samples. **(G, I)** one-way ANOVA with Tukey’s multiple comparison tests, results are shown by horizontal lines with asterisks below samples. For **(G, I)**, * p<0.05, ** p<0.01, *** p<0.001 and **** p<0.0001.

Six out of eleven ASOs significantly downregulated FOXP3 mRNA expression in CD4+CD25+ isolated healthy donor Tregs during overnight incubation, with an average 0.66 of residual FOXP3 mRNA expression (**Figure 2A**, **Supplementary Figure 1A**). FOXP3 downregulation was accompanied by significantly increased gene expression of IL-17 (**Figure 2B**). We also observed trends for decrease of TGFβ, CTLA-4 and IL-10 expression, and trends for increased expression of IL-1β and IL-2, although those changes were not significant (**Figure 2B**).

Very short 3.5 hours incubation of Tregs with 2.5 μM of ASOs FOXP3 resulted in substantially impaired Treg suppressive function, reaching significance for 5 of 6 ASOs: 15, 16, 21, 23 and 29 (**Figures 2C, D**, **Supplementary Figure 1B**). All ASOs that passed screening experiments and demonstrated significant effects on human Tregs during brief incubation targeted the 3’ untranslated region of FOXP3 mRNA (**Figure 1A**).

### 3.3 Effects of ASOs FOXP3 on human cancer samples

**Figure 3:**
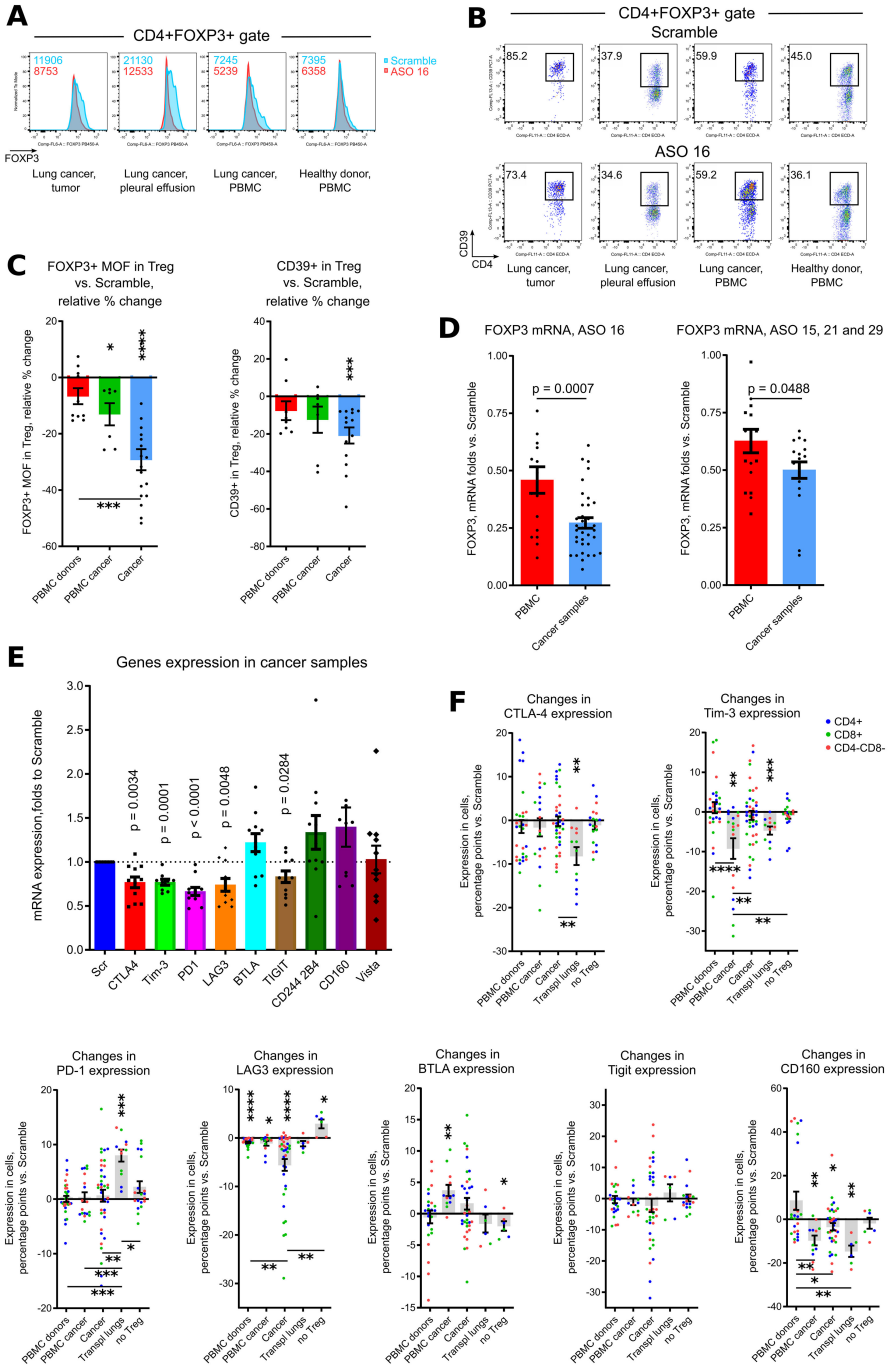
ASO FOXP3 effects on phenotype of Tregs and exhaustion of non-Treg cells. **(A-C)** same samples as in **Figures 2G–I**, were evaluated for FOXP3 median of fluorescence (MOF) and for CD39 expression in leftover Tregs, which preserved their FOXP3+ phenotype after 5 days of treatment. **(A)** representative histograms of FOXP3 expression in CD4+FOXP3+ Tregs, corresponding FOXP3 MOF values are shown in the left corners. **(B)** representative plots of CD39+ expression in CD4+FOXP3+ Tregs. **(C)** corresponding statistics for **(A, B)**, data calculated as in **Figures 2G, I**. **(D)** downregulation of FOXP3 mRNA by ASO16 (left) and by ASO 15, 21 and 29 (right) was compared for all healthy donors PBMC (from the experiments in **Figure 1F**) vs. all cancer samples (from the experiments in **Figure 2E**). **(E)** cells from the experiments in **Figure 2E** were evaluated for mRNA expression of exhaustion markers. **(F)** same samples as in **Figures 2G–I** were supplemented by additional samples, consisting of 3 transplant lungs and 6 Treg-depleted samples, and evaluated for the expression of 7 exhaustion markers. Data calculated as percentage points, i.e. “expression in ASO16 treated sample – expression in Scramble”. For this calculation, we used the averaged values for each sample evaluated in different flow cytometry panels, and comparison was calculated versus two (more often) or one independent Scramble controls. More data for each type of sample are shown in **Supplementary S2**, and more data with representative flow cytometry plots and with statistics for co-expression of exhaustion markers are shown in **Supplementary S3**. C, and F for PD-1, one sample Wilcoxon rank tests with median =0, only results with p<0.05 are shown using asterisks above samples. C, and F for PD-1, Kruskal-Wallis test with Dunn’s multiple comparison tests, results are shown by horizontal lines with asterisks below samples. **(D)** T-tests, two-tailed. **(E)** - one sample T-tests with mean = 1, only results with p<0.05 are shown. **(F)** all graphs except for PD-1, one sample T-tests with mean = 0, only results with p<0.05 are shown using asterisks above samples. **(F)** all graphs except for PD-1, one-way ANOVA with Tukey’s multiple comparison tests, results are shown by horizontal lines with asterisks below samples. *p<0.05, **p<0.01, ***p<0.001 and ****p<0.0001.

To study effects of Treg targeting for primary tumors, we studied 6 tumor samples, 7 malignant pleural effusion samples, 4 tumor-free distant lung samples, 4 lymph nodes and 5 PBMC samples from patients with lung cancer (12 out of 18 patients had adenocarcinoma), melanoma and mesothelioma. All 5 ASOs significantly downregulated FOXP3 mRNA expression in primary cancer samples (**Figure 2E**, **Supplementary Figure 1C**). As for healthy donors PBMC, targeting of FOXP3 was very specific and did not decrease the expression of other members of the FOXP family (**Figure 2F**). Using flow cytometry, we confirmed that ASO FOXP3 treatment resulted in decreased numbers of all cancer Tregs (**Figures 2G–I**, **Supplementary Figures 2A, B**) including intratumoral Tregs, analyzed separately (**Supplementary Figures 2A, B**). Leftover Tregs, which were still FOXP3+ after 5 days of treatment, decreased the amount of FOXP3 protein per cell and downregulated expression of the Treg associated marker, CD39 (**Figures 3A–C**, **Supplementary Figure 2C**). As a control, we studied 3 lung transplant samples, rejected for transplantation. Notably that those samples, although downregulated their Treg numbers, appeared to be less sensitive to the effect of ASO FOXP3 in comparison with distant tumor-free lung cancer samples and in comparison with other cancer samples (**Supplementary Figure 2**). At protein level, all cancer samples, were more sensitive to ASO FOXP3 than PBMC cancer samples (**Figures 2G, I**, **Supplementary Figures 2A, B**). At mRNA level, cancer samples were much more sensitive with their FOXP3 mRNA downregulation than healthy donors PBMC samples, independently of the candidate ASOs used (**Figure 3D**).

Additionally, the amount of FOXP3 protein per cell in leftover Tregs in cancer samples decreased more significantly than in PBMC samples (**Figure 3C**, **Supplementary Figure 2C**) and in transplant lungs (**Supplementary Figure 2C**). Therefore, human cancer Treg (intratumoral, pleural effusion, lymph nodes and even distal tumor-free cancer lungs) were more sensitive to ASO FOXP3, in comparison with peripheral Tregs, at both FOXP3 mRNA and protein levels.

Downregulation of FOXP3 in cancer Tregs was accompanied by significantly decreased mRNA expression of five out of nine tested exhaustion markers: CTLA-4, Tim-3, PD-1, LAG-3 and TIGIT (**Figure 3E**) in the endogenous T cells. At the protein level, expression of exhaustion markers on T cells varied for different markers in different samples (**Figure 3F**, **Supplementary Figure 3A**). Analysis of co-expression of two and more exhaustion markers shows that combination of BTLA and CD160 with or without LAG-3, conversely to other markers, show the maximum of downregulation in cancer samples (**Supplementary Figure 3B**). We also tested the effects of ASO FOXP3 on six Treg depleted samples, two of which were healthy donors PBMC and four were cancer tumors, lymph nodes and pleural effusion. Notably that those “no Treg” samples demonstrated almost no changes in their expression of exhaustion markers and even slightly increased expression for some of them (**Figure 3F**, **Supplementary Figure 3B**). This observation suggests that downregulation of exhaustion markers is related with an effects of ASO FOXP3 on Tregs rather than with a direct effects of ASO FOXP3 on other cells.

**Figure 4:**
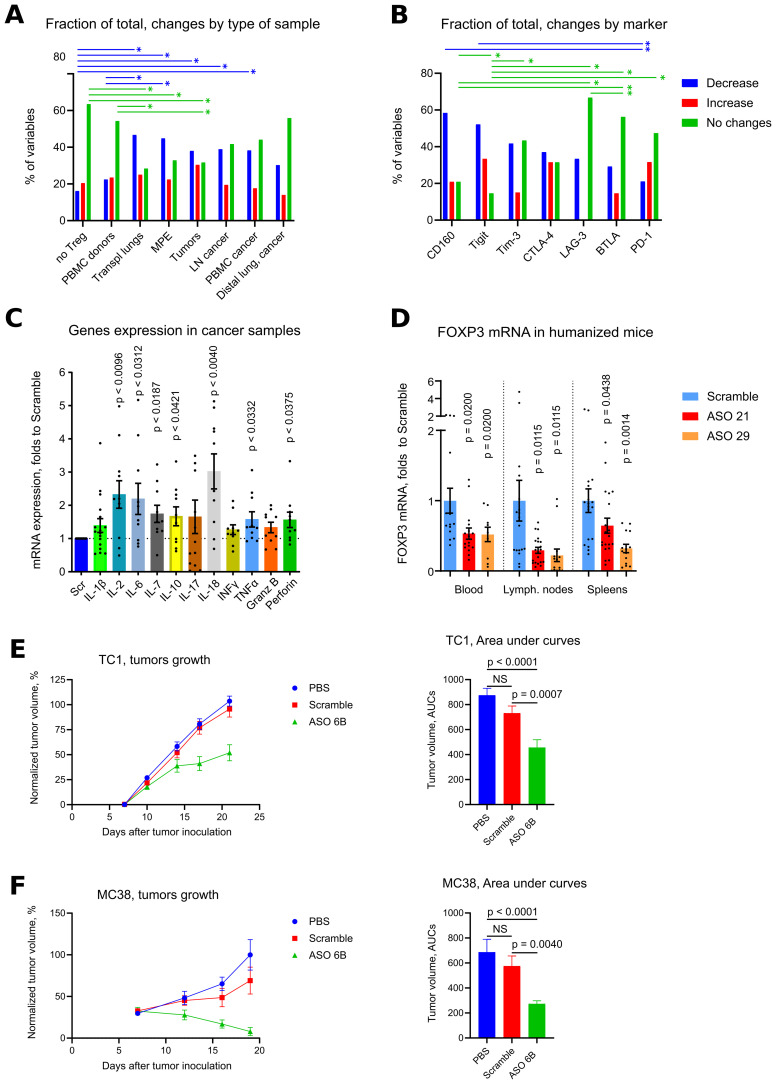
Overview of exhaustion *in vitro*, effects of human and murine ASO FOXP3 *in vivo*. **(A)** same samples as in **Figure 3F**, were used for quantitative evaluation of in each exhaustion marker within each sample and within cellular subsets, comprising of CD4+ T cells, CD8+ T cells or CD4-CD8- non-T cells. A decrease of expression for 3% and less vs. Scramble counted as one event in “decrease” category, changes within -3% to +3% were counted as events in “no changes” category, and an increase of expression for 3% and more was counted as one event in “increase” category. Then number of events were summarized within each type of samples **(A)** or within each type of exhaustion marker **(B)** except for “no Treg” samples. To visualize groups for a direct comparison, corresponding fraction of events within each group is shown in **(A, B)**, such as % of “decrease” + % of “increase” + % of “no change” events within “no Treg” group is equal 100%. Similarly, % of “decrease” + % of “increase” + % of “no change” events within “BTLA” group is equal 100%. **(C)** cells from the experiments in **Figure 2E** were evaluated for mRNA expression of cytokines. **(D)** humanized mice hu-PBMC-NSG, 5 mice per group in 2 experiments (20 mice total) were treated with Scramble controls or human ASO FOXP3 by i.p. injections 50 mg/kg daily x4 times. Then mouse blood, spleens and LNs were collected and evaluated by qPCR. FOXP3 mRNA expression are shown. **(E)** 165 mice in 7 experiments were treated with PBS, Scramble or ASO 6B 50 mg/kg daily since day 7^th^ after tumor inoculation. Left, data of tumors growth were 0–100 normalized (detailed in Methods) within each experiment, then combined. Right, area under curves of normalized tumor growths were calculated and compared. **(F)** 65 mice in 3 experiments were treated as in **(E)** Left, data of tumor growth were 0–100 normalized (detailed in Methods) within each experiment, then combined. Right, area under curves of normalized tumor growths were calculated and compared. Non-normalized data of tumor growths from 1 TC1 and 1 MC38 experiment are shown in **Supplementary Figures 4A, B**. **(A, B)**. Chi-square tests (both are p<0.0001), followed by with Pairwise Z-Tests with Bonferroni corrections, only results with p<0.05 for paired comparisons are shown as horizontal lines with asterisks, lines are colored according to the corresponding changes in expression. SPSS did not provide with exact p values for those tests. **(C, D)** - one sample T-tests with mean =1, only results with p<0.05 are shown. **(E, F)** – Kruskal-Wallis test with Dunn’s multiple comparison test. *p<0.05, **p<0.01, NS - not significant.

In order to compare behavior of all exhaustion markers between samples, we calculated quantitative changes in each marker within each sample and within 3 cellular subsets (CD4+ T cells, CD8+ T cells or CD4-CD8- non-T cells), such a decrease of expression for 3% and less vs. Scramble counted as one event in “decrease” category, changes within -3% to +3% were counted as events in “no changes” category, and an increase of expression for 3% and more was counted as one event in “increase” category (**Figure 4A**). As expected, Treg depleted samples and healthy donors PBMC were the least sensitive with their changes in exhaustion markers due to targeting of Tregs. Cancer samples, and, surprisingly, transplant lungs samples, were in the group with more often “decreases” than “no changes” observed. PBMC lung cancer samples and tumor free distal lung samples had moderately prevalent “no changes” reaction followed by “decrease” reaction (**Figure 4A**). We also compared the behavior of different exhaustion markers across all (except for “no Tregs”) samples, using the same approach in calculation as above. CD160 and Tigit, followed by Tim-3, demonstrated the maximum rates of decrease, while LAG-3, BTLA and PD-1 had the prevalent reaction “no change” (**Figure 4B**).

Overall, expression of exhaustion markers mostly decreased at mRNA and protein levels in cancer samples, but not in Treg depleted samples and not in PBMC from healthy donors.

Expression of exhaustion markers per se does not mean an exhausted status of corresponding T cells, since those markers can be upregulated in activated cells (49–53). To clarify this point, we evaluated expression of inflammatory cytokines in cancer samples, and found that mRNA expression of most of them were upregulated as a result of ASO FOXP3 targeting of Tregs. Differences were significant for 7 of 11 cytokines: IL-2, IL-6, IL-7, IL-10, IL-18, TNF-α and Perforin-1 (**Figure 4C**). Therefore, downregulation of exhaustion markers was accompanied by enhanced expression of inflammatory cytokines, indicating that ASO FOXP3 targeting of Tregs at least partially reversed the exhausted phenotype of intratumoral T cells. To conclude, ASO FOXP3 targeting of Tregs in cancer samples was accompanied by decreased number of Tregs, reduced mRNA FOXP3 expression, lower amounts of FOXP3 in Tregs that still expressed FOXP3, decreased CD39 expression in those Tregs, and downregulation of exhaustion markers on non-Treg cells, along with their enhanced expression of inflammatory cytokines.

### 3.4 Effects of human ASO FOXP3 *in vivo*

We injected ASO21 and ASO29 into humanized mice hu-PBMC-NSG, and found that 4 daily injections of ASO FOXP3 at the dose 50 mg/kg, efficiently downregulated human FOXP3 mRNA expression in the blood, LNs and spleens (**Figure 4D**) of those mice, confirming that human ASOs were efficient *in vivo*.

### 3.5 Effects of murine ASO FOXP3 on tumors

To study effects of ASO FOXP3 on tumors *in vivo*, we treated mice with a murine analogues of ASO FOXP3, called ASO 6B. Mice in two tumor models, TC1 and MC38, received PBS, Scramble control or ASO 6B 50 mg/kg i.p. daily for 14 days. We observed significant inhibition of tumor growth in both models (**Figures 4E, F**, **Supplementary Figures 4A, B** for two individual experiments). Moreover, 22% of TC1 tumors and 13.6% of MC38 tumors were completely resorbed. At the end of experiments, many TC1 tumors and most MC38 tumors treated with ASO 6B were small, comprised of necrotic and connective tissues and had few viable infiltrating cells.

**Figure 5:**
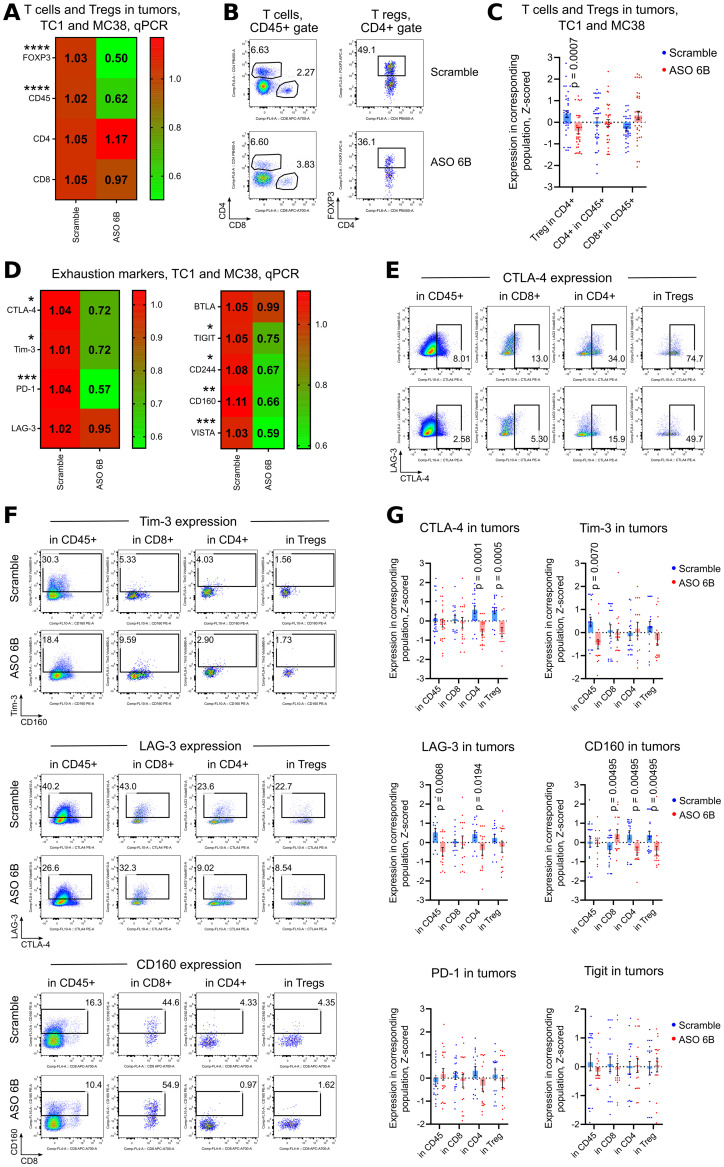
Expression of exhaustion markers in TC1 and MC38 tumors. Mice in two tumor models, TC1 and MC38 were treated as in **Figures 4E, F**. **(A, D)** 18 TC1 tumors and 16 MC38 tumors were harvested at day 14^th^ after tumor inoculation. MC38 tumors, due to the small size, especially in ASO treated groups, were combined together as 2–3 tumors per one RNA sample. Heatmaps of qPCR data, representing an average values, with corresponding statistics shown for **(A)** FOXP3 and T cells; **(D)** for expression of exhaustion markers. Color keys for heatmaps shown on the right of each heatmap. Detailed qPCR data are shown in **Supplementary Figure 6C**. **(B, C, E-G)**. 26 TC1 tumors and 35 MC38 tumors were harvested at day 14th after tumor inoculation. MC38 tumors, due to the small size, especially in ASO treated groups, were combined together as 2–3 tumors per one flow cytometry sample. **(B)** representative plots of T cells (left) and Treg (right) staining in TC1 tumors and **(C)** corresponding statistics with Z-scored flow cytometry data. More flow cytometry data are shown in **Supplementary Figures 6A, B**. **(E, F)** representative flow cytometry plots of **(E)** CTLA-4 expression and **(F)** Tim-3, Lag-3 and CD160 expression. **(G)** corresponding statistics with Z-scored flow cytometry data. More representative plots are shown on **Supplementary Figures 7A, B**. Non-normalized flow cytometry data with Tregs and with expression of all evaluated exhaustion markers for one TC1 and one MC38 experiment are shown on **Supplementary Figures 4C**, **5**. **(A, C, D, G)** - multiple T-tests FDR Benjamini, Krieger and Yekutieli, with Q = 5%. Only differences with p<0.05 are shown *p<0.05, **p <0.01, ***p<0.001, ****p<0.0001.

**Figure 6:**
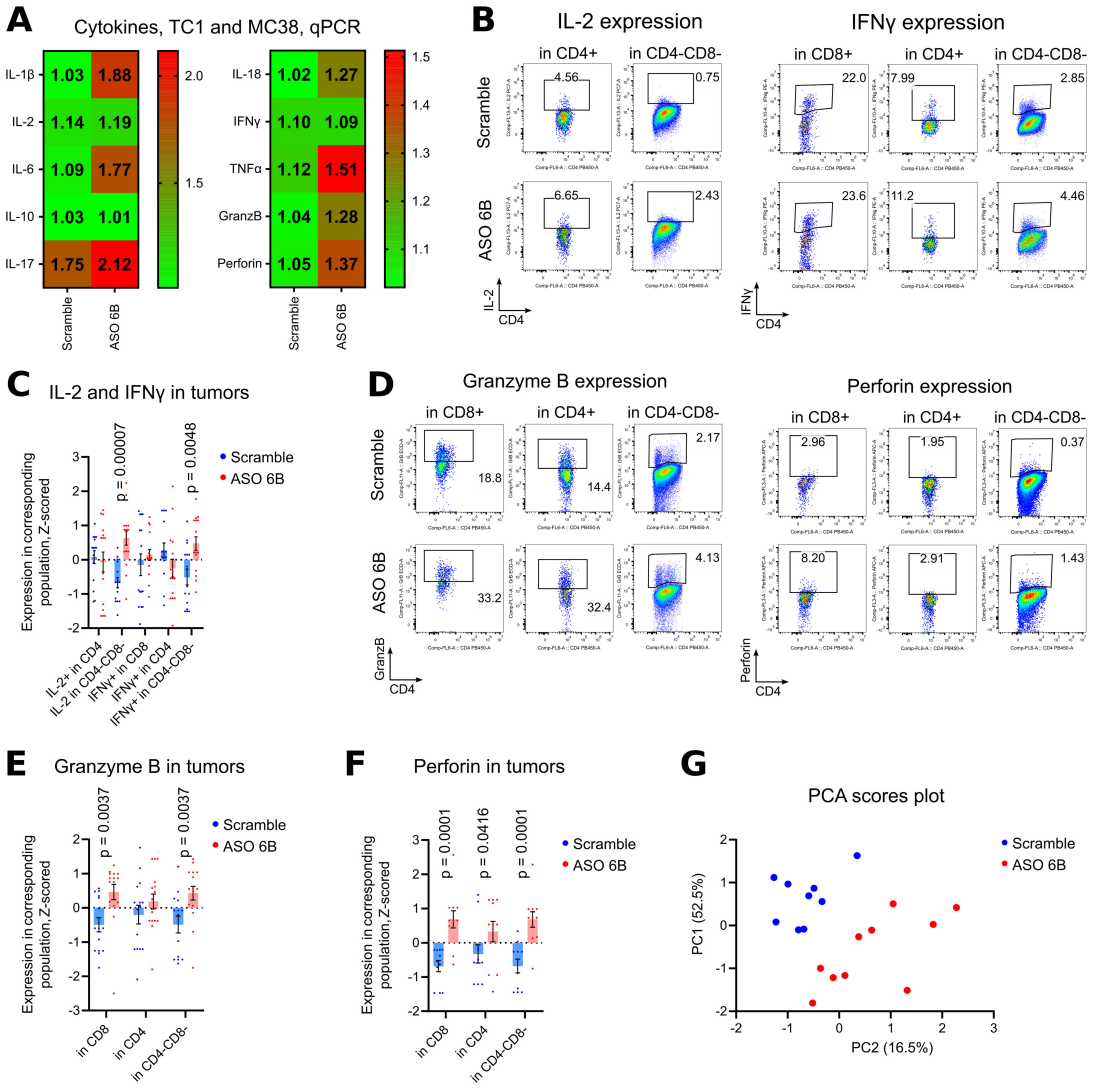
Cytokines production in TC1 and MC38 tumors. **(A)** 18 TC1 tumors and 16 MC38 tumors as in **Figures 5A, D**, we evaluated by qPCR for cytokines expression. Heatmaps of qPCR data, representing an average values, with corresponding statistics shown. Color keys for heatmaps shown on the right of heatmap. Detailed qPCR data are shown in **Supplementary Figure 6D**. **(B-F)** 19 TC1 and 12 MC38 tumors as in **Figures 5B, C, E–G**, were stimulated for 4 h with PMA/ionomycin with Monensin, then evaluated for cytokines expression. Flow cytometry plots show representative data with staining for **(B)** IL-2 and IFNγ; **(D)** Granzyme B and Perforin. **(C, E, F)**. same tumors, statistics for Z-scored flow cytometry data. More flow cytometry plots and statistics are shown in **Supplementary Figures 7C**. **(D)** Non-normalized flow cytometry data with expression of evaluated cytokines for one TC1 and one MC38 experiment are shown on **Supplementary Figure 5**. **(G)** PCA analysis was performed with Z-scored flow cytometry data from the same tumors as **(C-F)** and as in **Figures 5C, G**. Variables include Treg numbers in CD4+ subset, expression of exhaustion markers in different subsets of cells, and production of inflammatory cytokines by those cells. Two components were extracted, based on the eigenvalues over 1 criterion and the scree plot. Direct Oblimin rotation was applied. More details are reported in **Supplementary Table 2**. Scores plot of PC1 vs. PC2 is shown. **(A, C, E, F)**. multiple T-tests FDR Benjamini, Krieger and Yekutieli, with Q = 5%. Only differences with p<0.05 are shown.

To overcome this problem and to study intratumoral Tregs and immune response *in situ*, we harvested tumors midway through our experiments, after 1 week of treatment. We observed significant decrease of FOXP3 mRNA expression within tumors and, correspondingly, significantly less intratumoral Tregs using flow cytometry (**Figures 5A, C**, **Supplementary Figures 6A, C**). The remaining FOXP3+ intratumoral murine Treg that still expressed FOXP3 after treatment, had no significant differences in their amount of FOXP3 protein per cell, in contrast to the *in vitro* human data (**Supplementary Figure 6B**). Overall, immune infiltration (CD45 mRNA) decreased, with no clear trends for CD4+ and CD8+ T cells (**Figures 5A, C**, **Supplementary Figure 6C**). Targeting of intratumoral Treg *in vivo* was associated with significantly downregulated expression of exhaustion markers, 7 out of 9 for mRNA expression and 4 out of 6 for protein expression (**Figures 5D–G**, **Supplementary Figures 6C, 7A, B**). As an indirect confirmation of alleviated exhaustion along with decreased Treg suppression, we observed trends for increased expression of mRNA for most inflammatory cytokines (**Figure 6A**). Moreover, intratumoral T cells in ASO FOXP3 treated mice produced more Perforin and Granzyme B, while CD4-CD8- non T cells had increased IL-2 and IFN-γ production (**Figures 6B–F**, **Supplementary Figures 6D**, **7C, D**), which confirms our suggestion that targeting of intratumoral Tregs with ASO FOXP3 can at least partially reverse an exhausted phenotype of intratumoral T cells.

Finally, we performed PCA analysis of flow cytometry data for tumor infiltration leukocytes to assess how the number of intratumoral Tregs, expression of exhaustion molecules, and cytokine production variables clustered. Two components were extracted, the first component accounted for 52.5% of the variance and the second component accounted for 16.5% of the variance. The **Supplementary Table 2** displays the items and component loading for the rotated components. PCA plot reveals two clearly separated groups, according to ASO FOXP3 treatment (**Figure 6G**). Expression of exhaustion markers (LAG-3 and Tim-3, PD-1), followed by number of Tregs, loaded into component 1, all with positive values. The most influential variables in component 2 were IFN-γ expression, loaded with positive value, and PD-1 expression on CD4+ with a negative value (**Supplementary Table 2**).

Overall, treatment of ASO FOXP3 *in vivo* resulted in significantly decreased Treg numbers and FOXP3 mRNA expression, along with decreased expression of exhaustion markers on infiltrating intratumoral T cells, which corresponded with increased production of Granzyme and Perforin.

### 3.6 Systemic and local effects of murine ASO FOXP3 in tumor bearing mice

**Figure 7:**
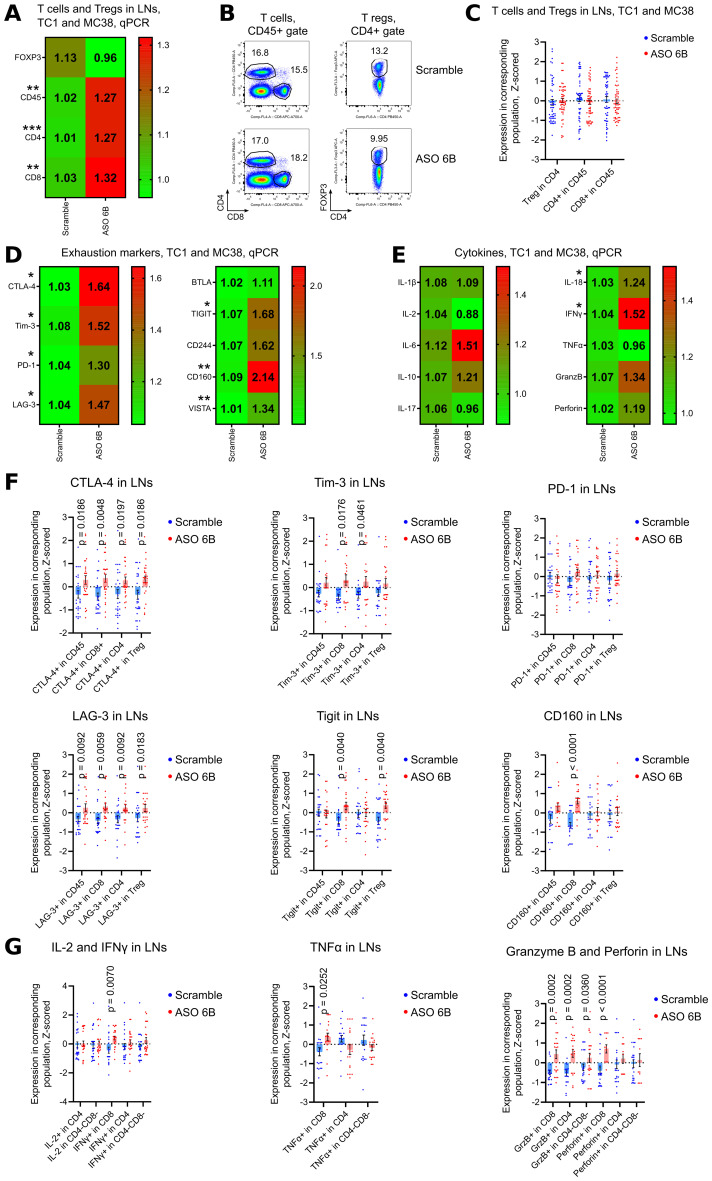
Analysis of draining lymph nodes of ASO FOXP3 treated TC1 and MC38 tumor bearing mice. **(A, D, E)** 19 LNs from TC1 tumors mice and 10 LNs from MC38 mice as in **Figures 5A, D**, were evaluated by qPCR. Heatmaps of qPCR data, representing an average values, with corresponding statistics shown for **(A)** FOXP3 and T cells; **(D)** for the expression of exhaustion markers and **(E)** for the expression of cytokines mRNAs. Color keys for heatmaps shown on the right of each heatmap. Detailed qPCR data are shown in **Supplementary Figures 8B–E**. **(B, C, F, G)** 28 LNs from TC1 tumors mice and 28 LNs from MC38 mice as in **Figures 5B, C**, were evaluated by flow cytometry. **(B)** representative plots of T cells (left) and Treg (right) staining in TC1 LNs and **(C)** corresponding statistics with Z-scored flow cytometry data. **(F)** expression of exhaustion markers in TC1 and MC38 LNs, statistics for Z-scored flow cytometry data. **(G)** LNs cells were stimulated for 4 h with PMA/ionomycin with Monensin. Statistics for cytokines production in LNs, Z-scored flow cytometry data. Detailed flow cytometry data are shown in **Supplementary Figures 8A, F, G, 9A–I**. **(A, C-G)**, multiple T-tests FDR Benjamini, Krieger and Yekutieli, with Q = 5% * - p value <0.05, **p <0.01, ***p<0.001.

In tumor draining LNs, expression of FOXP3 mRNA and numbers of Tregs were similar between Scramble and ASO 6B treated mice (**Figures 7A–C**, **Supplementary Figures 8A, B**). Expression of CD45, CD4 and CD8 mRNA increased in ASO FOXP3 treated group, but percent of T cell subsets in CD45+ gate did not change (**Figures 7A–C**, **Supplementary Figure 8B**). Most of the exhaustion markers were upregulated in ASO 6B treated group when compared with Scramble (**Figures 7D, F**, **Supplementary Figures 8C, D, F, G**, **9A–D**). Levels of most inflammatory cytokines in draining LNs were also increased or demonstrated trends to be increased (**Figures 7E, G** **Supplementary Figures 8E**, **9E–I**).

**Figure 8:**
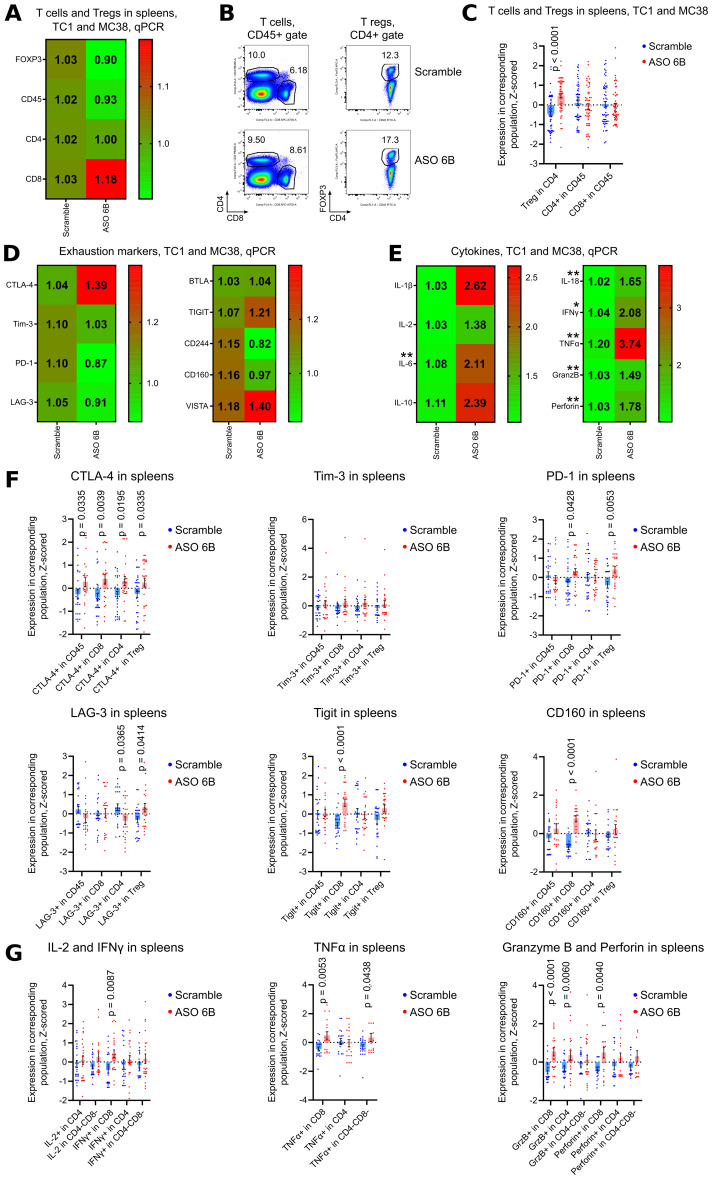
Analysis of spleens of ASO FOXP3 treated TC1 and MC38 tumor bearing mice. **(A, D, E)** 19 spleens from TC1 tumors mice and 10 spleens from MC38 mice as in **Figure 5A**, were evaluated by qPCR. Heatmaps of qPCR data, representing an average values, with corresponding statistics shown for **(A)** FOXP3 and T cells; **(D)** for the expression of exhaustion markers and **(E)** for the expression of cytokines mRNAs. Color keys for heatmaps shown on the right of each heatmap. Detailed qPCR data are shown in **Supplementary Figures 10B–E**. **(B, C, F, G)** 28 spleens from TC1 tumors mice and 28 spleens from MC38 mice as in **Figures 5B, C**, were evaluated by flow cytometry. **(B)** representative plots of T cells (left) and Treg (right) staining in TC1 spleens and **(C)** corresponding statistics with Z-scored flow cytometry data. **(F)** expression of exhaustion markers in TC1 and MC38 spleens, statistics for Z-scored flow cytometry data. **(G)** splenocytes were stimulated for 4 h with PMA/ionomycin with Monensin. Statistics for cytokines production in the spleens, Z-scored flow cytometry data. Detailed flow cytometry data are shown in **Supplementary Figures 10A, F, G, 11A–I**. **(A, C-G)**, multiple T-tests FDR Benjamini, Krieger and Yekutieli, with Q = 5% * - p value <0.05, **p <0.01.

To analyze systemic effects of ASO FOXP3 targeting of Tregs in tumor bearing mice, we performed immune profiling of the spleens. Numbers of Tregs were increased in the spleens of ASO FOXP3 treated mice without the changes in FOXP3 mRNA expression (**Figures 8A–C**, **Supplementary Figures 10A, B**). While draining LNs showed an apparent increase of mRNA expression of exhaustion molecules, spleens showed no changes in their mRNAs but, as in LNs, spleens had significantly increased expression of some exhaustion markers by flow cytometry (**Figures 8D–F**, **Supplementary Figures 10C, D, F, G**, **11A–D**). Those effects were accompanied by upregulated mRNA expression and by increased production of the most inflammatory cytokines, especially by CD8+ T cells (**Figures 8E, G**, **Supplementary Figures 10E**, **11E–I**).

### 3.7 Effects of ASO FOXP3 targeting on T cell exhaustion *in vivo*

We observed that in draining LNs (local immune response) and in the spleens (systemic immune response) of tumor bearing mice the expression of exhaustion markers *in vivo* had the same trend as expression of inflammatory cytokines, i.e. both were increased by ASO FOXP3 targeting of Tregs (**Figures 7**, **8**). Those data are in contrast to tumor data, where intratumoral expression of exhaustion markers decreased along with targeting of Treg, but expression of inflammatory cytokines increased (**Figures 5**, **6**). This discrepancy might be explained by significantly different initial levels of expression of exhaustion molecules in tumors vs. LNs and spleens (**Supplementary Figures 12A–F**). Therefore, since levels of all exhaustion markers in tumors were high, we suggest that only intratumoral T cells, but not T cells in LNs and spleens, had the “true” exhausted phenotype, which was partially reversed when suppressive intratumoral environment was disrupted by ASO FOXP3 targeting of Tregs.

### 3.8 Phenotype of Treg after FOXP3 downregulation

**Figure 9:**
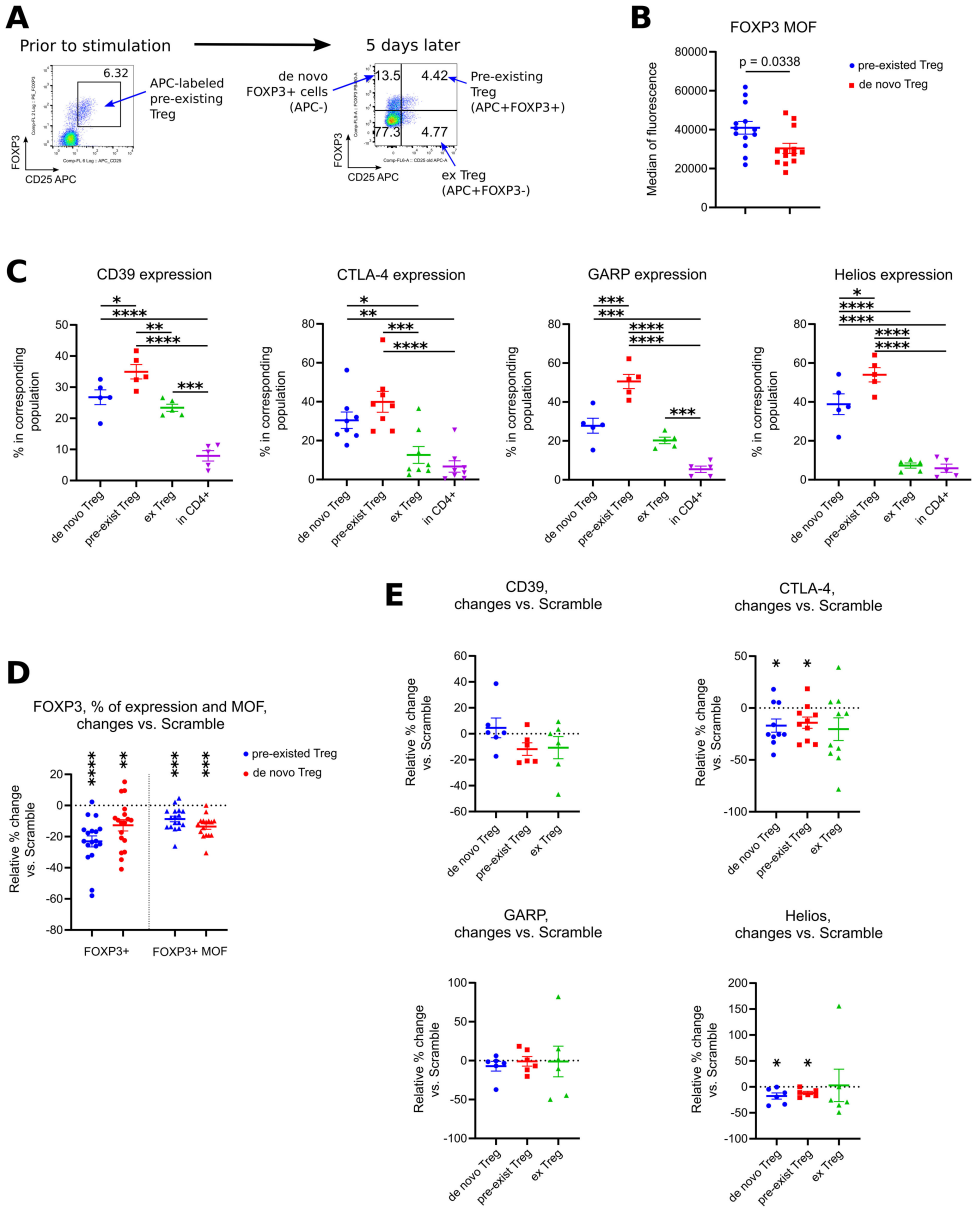
Analysis of phenotype of Tregs with downregulated FOXP3 expression. **(A-E)** PBMC from 5 different donors in 4 experiments were incubated with CD25 APC antibodies for 40 min, then washed and treated with 1.5 μM of Scramble or ASO FOXP3 15, 21, 23 or 29 in experimental conditions as in **Figures 1B–E**, for 5 days. After treatment, cells were evaluated by flow cytometry. **(A)** co-expression of CD25 APC vs. FOXP3 allowed us to distinguish 4 different populations of cells: APC-FOXP3+ “*de novo*” Tregs, APC+FOXP3+ “pre-existed” Tregs, APC+FOXP3- exTregs and CD4+APC-FOXP3- conventional CD4+ T cells. **(B)** Median of fluorescence (MOF) for FOXP3 protein expression in all “pre-existed” vs. all “*de novo*” Tregs. Each PBMC sample was tested with 1–3 ASOs and 1–2 Scrambles. **(C)** expression of CD39, CTLA-4, GARP and Helios in corresponding subsets of cells. Data for ASO FOXP3 treated cells shown, data for Scramble treated cells had the same trends. **(D)** changes in FOXP3+ expression (left) and in FOXP3 MOF expression (right) in “pre-existed” and in “*de novo*” ASO FOXP3 treated Tregs in comparison with Scramble treated Tregs. Data calculated as percent of changes over control: 100*((current ASO result – Scramble result)/Scramble result). **(E)** changes in expression of CD39, CTLA-4, GARP and Helios in ASO FOXP3 treated cells in comparison with Scramble treated cells, calculated as in D. **(B)**, Mann-Whitney test. **(C)** one-way ANOVA with Tukey’s multiple comparison tests, results are shown by horizontal lines with asterisks above samples. **(D, E)** except for GAPR, one sample T-tests with mean =0. **(E)** GARP - one sample Wilcoxon rank test with median =0. **(D, E)**. only results with p<0.05 are shown using asterisks above samples. *p<0.05, **p<0.01, ***p<0.001 and ****p<0.0001.

To study if Treg with downregulated FOXP3 convert into conventional T cells or rather keep their “Treg-like” phenotype, we performed two experiments: *in vitro* with pre-labeled human Treg (**Figure 9A**) and *in vivo* with RAG1-/- mice, injected with a mix of Thy1.2 Tregs and Thy1.1 CD4+CD25- conventional T cells (**Supplementary Figures 13A–D**). Both strategies allowed us to track changes in phenotype of affected Tregs, although only for few days. In both models, we observed two processes of opposite directions, occurring independently of ASO treatment: a) some conventional T cells upregulated their FOXP3 expression, and we called them “*de novo*” Tregs; b) some Treg lose their FOXP3 expression, so we called them “ex Treg”. Therefore, we compared phenotype of “pre-existed Tregs”, “*de novo* Tregs” and “ex Tregs”, but each time those populations comprised a mixture of cells naturally undergoing with corresponding changes in their FOXP3 levels and with the cells with forcefully downregulated FOXP3 by ASO.

In humans, “*de novo*” Treg, in comparison with “pre-existed” labeled Treg, had lower levels of FOXP3 protein per cell (**Figure 9B**), but their overall phenotype was similar to Treg with lower expression of CD39, GARP and Helios and with a trend to lower expression of CTLA-4 (**Figure 9C**). Similarly, ex Treg gradually lose their expression of Treg associated markers, but with a different rate. Thus, CD39 and GARP expression was downregulated moderately, while CTLA-4 and Helios expression almost reached the levels of their expression in conventional T cells (**Figure 9C**). Next, we compared phenotype of Treg-related groups in ASO FOXP3 treated vs. Scramble samples to see an additional effect of forcefully downregulated FOXP3 on Treg phenotype. ASO FOXP3 downregulated FOXP3 expression in both types of FOXP3+ cells almost equally, resulting with decreased % of Tregs in both, “pre-existed” Treg subset as well as in “*de novo*” Treg subset (**Figure 9D**). Another effect was a decrease of FOXP3 protein per cell in Tregs that were still FOXP3-positive, the same effect that we observed with all human samples in the previous experiments. Both, “*de novo*” Tregs and “pre-existed” Tregs, had similarly less FOXP3 protein levels in ASO FOXP3 treated samples in comparison with Scramble treated samples (**Figure 9D**). ASO FOXP3 treatment had no apparent effect in expression of all tested markers in exTreg subset (**Figure 9E**), but slightly decreased expression of CTLA-4 and Helios in “*de novo*” Tregs and in “pre-existed” Tregs (**Figure 9E**).

In mice, ex Tregs appeared to have less CD39 and CTLA4 expression in comparison with stable Tregs, as in humans, but we have not seen any clear additional effects of ASO FOXP3 on the expression of those markers (**Supplementary Figure 13E**). Additionally, ex Tregs had lower rate of divisions *in vivo* in comparison with stable Tregs.

Overall, our data show that ex Tregs have an intermediate phenotype, more or less resembling stable Tregs with lower expression of Treg associated markers, but we have not observed any apparent signs of their conversion into conventional T cells, and ASO FOXP3 have relatively weak effect in promoting further downregulation of Treg associated markers. To further evaluate risks of autoimmunity and inflammation, we evaluated the rate of divisions of conventional CD4+ T cells and RAG1-/- cells *in vivo*, and found no increase in ASO FOXP3 treated group (**Supplementary Figure 13D**). Similarly, ASO FOXP3 treated mice in TC1 model had no apparent histologic evidences of autoimmunity or inflammation in their tissues (**Supplementary Figure 14A**), which was supported by an absence of CD25 upregulation in non-Treg subsets (**Supplementary Figure 14B**). We observed a moderate increase of CD69 expression in CD4+ and CD8+ cells in ASO FOXP3 treated TC1, but not in MC38 mice (**Supplementary Figure 14B**). That increase had no corresponded clonal activation, evaluated as a rate of cellular divisions *in vivo* (**Supplementary Figure 14C**). To conclude, we have not observed any signs of apparent autoimmunity or inflammation, either from ex Treg subsets or from the non-Treg cells, *in vivo* and *in vitro*, in tumor bearing mice or in RAG1-/- mice.

## 4 Discussion

Regulatory T cells play an important role in suppressing anti-tumor immune responses, and hence, their targeting is an important part of strategies to enhance anti-tumor immunity and increase the effectiveness of immunotherapy (8, 54–56). One of the major limitations of current Treg-targeted therapies is the lack of selectivity, resulting in concurrent depletion of systemic Tregs and anti-tumoral effector T cells (54). Use of antisense oligonucleotides targeting FOXP3 is a novel technology that overcomes the problem of the absence of a unique superficial Treg marker to target. As FOXP3 is a master regulator of Treg development and function, its downregulation by ASO is reasonably assumed to disrupt Treg mediated suppression in tumors. Recently, AstraZeneca and Ionis Pharmaceuticals have used an older generation of ASO technology to target FOXP3 for cancer models and shown limited efficacy in their preclinical studies. The sole publication describing ASO studies with the IONIS reagent (57) studied effects on human and murine *ex vivo* expanded Tregs of unknown purity, *in vitro* converted iTregs, and activated Helios+CD4+ cells, but critically, no data were provided concerning ASO effects on primary Treg cells, except for qPCR analysis of FOXP3 in humanized mice. Modest *in vivo* anti-tumor effects were shown in some Treg dependent models but not in others, e.g., no effects were seen in MC38 colon cancer tumor model, while in our study, ASO treatment in the MC38 tumor model was as efficient as in TC1 tumors. AstraZeneca began a Phase 1a/b clinical trial (NCT04504669) with that older generation of ASO to target FOXP3.

In the current study, we used a new, highly improved type of ASO. In contrast to previous ASO, FOXP3 AUMsilence ASO do not require delivery agents, and are capable of highly specific RNA silencing of previously “undruggable” targets (32–38). ASO first binds to the RNA target using highly specific Watson-Crick base pairing (58) and recruits endogenous RNase H that recognizes the mRNA/ASO hybrid and cleaves RNA within the hybrid (59, 60). After cleavage, fragmented RNA is degraded by nucleases and ASOs are recycled, increasing efficiency and lowering the dose required by allowing one ASO to degrade many copies of mRNA (61, 62). Also, unlike siRNAs that are only processed in the cytoplasm, AUMsilence ASOs can shuttle into the nucleus and silence nuclear RNA (63). Compared to non-modified previous generation ASO, AUMsilence ASO modification has an enhanced stability inside cells, due to resistance to nucleases (59, 60, 64) and in the bloodstream (40), and do not activate Toll-like receptor signaling (39). AUMsilence ASOs have been used to target T cells, neurons, and stem cells *in vitro* and *in vivo*, without triggering toxicity or immune responses (32–38, 65). Therefore, this 3rd generation of ASO FOXP3 overcomes many challenges in RNA silencing, which justified our choice to use them for targeting FOXP3 and hence Tregs.

We reported effects of ASO FOXP3 on primary human Tregs, and it is important to stress that those Tregs were not expanded *ex vivo* and were not converted *in vitro* from CD4+ T cells. Very short (3.5 hours) incubation of human Tregs with ASO FOXP3 was sufficient to decrease Treg suppressive function almost twice (66.4%) that seen in Scramble control, and overnight (16–18 hours) treatment was enough to downregulate FOXP3 mRNA expression to 0.66 of Scramble. To our knowledge, we are the first to report the effects of FOXP3 targeting on intratumoral Treg in clinical samples from patients with adenocarcinoma, melanoma, mesothelioma and squamous cell carcinoma, and we showed a 65% decrease on average of Treg mRNA and a 60% decrease of Treg numbers in the CD45+ subset.

Successful Treg targeting, as a part of anti-tumor therapy, raises legitimate concerns of safety, as Tregs are critical regulators of immune homeostasis, preventing autoimmunity and an excess of inflammation (8, 54–56). In the current study, we showed that *in vitro* peripheral blood Tregs are much less sensitive to the effects of human ASO FOXP3 in comparison with intratumoral Tregs. Moreover, we confirmed those observations *in vivo*; thus, only intratumoral Tregs but not draining LN and spleen Tregs were affected by murine ASO FOXP3 treatment. While the mechanism of that enhanced sensitivity of intratumoral Treg to ASO FOXP3 is the subject of ongoing investigation, we hypothesize that it may be related to the relatively high turnover of FOXP3 in intratumoral Treg. We have previously reported that intratumoral Tregs from lung cancer patients had significantly enhanced suppressive function and ongoing high turnover of FOXP3. They had almost twice as much FOXP3 protein/cell and about 2.5 times more FOXP3 mRNA in comparison with extratumoral Tregs (30). We also found upregulation of FOXP3 protein reflected heightened production of fresh FOXP3 protein from FOXP3 mRNA, but not enhanced stability of pre-existed FOXP3 (30). Therefore, the high sensitivity of intratumoral, but not conventional Treg to ASO FOXP3 is most probably the result of ongoing and high level of production of FOXP3, and is analogous to the higher sensitivity of cancer cells to chemotherapy in comparison with normal cells, due to their excessive rate of divisions. Since data of intratumoral Treg biology are relatively scarce, it is currently unknown what factors are responsible for such high FOXP3 turnover.

Exhaustion of intratumoral T cells (66, 67) is one of the main obstacles of anti-cancer therapy, and the most promising approach is the use of checkpoint inhibitors. However, the efficiency of checkpoint inhibitors in solid tumors is relatively low, which may be related with innate and acquired resistance (67–69). In our current study, we showed that targeting of intratumoral Tregs with ASO FOXP3 was accompanied by significant reduction in expression of exhaustion molecules on the tumor T cells, *in vivo* in tumor models and *in vitro* in human primary tumor samples. This reduction was associated with increased expression of inflammatory cytokines, especially granzyme B and perforin, both of which are known to be important for anti-tumor immune responses. Moreover, we showed that in the absence of Treg, ASO FOXP3 did not change expression of exhaustion molecules by intratumoral T cells. These observations suggest that intratumoral Tregs are important to the initiation and maintenance of the exhausted phenotype of tumor-associated T cells, and therefore Treg targeting with ASO FOXP3 may overcome resistance to checkpoint inhibitors therapies, leading to enhanced anti-tumor responses (70, 71). It will be important to study the conditions and mechanisms responsible for Treg-induced T cell exhaustion in future studies.

To conclude, we show that targeting of intratumoral Tregs using ASO FOXP3 monotherapy results in enhanced immune responses to solid tumors without inducing systemic hyperinflammation, and we propose may have benefits when used in combination with checkpoint inhibitors or with CAR-T cell therapy.

## Data Availability

The raw data supporting the conclusions of this article will be made available by the authors, without undue reservation.

## References

[1] Sakaguchi S Miyara M Costantino CM Hafler DA. FOXP3+ regulatory T cells in the human immune system. Nat Rev Immunol. (2010) 10:490–500. doi: 10.1038/nri2785 20559327

[2] Rudensky AY. Regulatory T cells and foxp3. Immunol Rev. (2011) 241:260–8. doi: 10.1111/j.1600-065X.2011.01018.x PMC3077798 21488902

[3] Takeuchi Y Nishikawa H. Roles of regulatory T cells in cancer immunity. Int Immunol. (2016) 28:401–9. doi: 10.1093/intimm/dxw025 PMC4986235 27160722

[4] Huang L Guo Y Liu S Wang H Zhu J Ou L. Targeting regulatory T cells for immunotherapy in melanoma. Mol Biomed. (2021) 2:11. doi: 10.1186/s43556-021-00038-z 34806028 PMC8591697

[5] Van Damme H Dombrecht B Kiss M Roose H Allen E Van Overmeire E. Therapeutic depletion of CCR8(+) tumor-infiltrating regulatory T cells elicits antitumor immunity and synergizes with anti-PD-1 therapy. J Immunother Cancer. (2021) 9. doi: 10.1136/jitc-2020-001749 PMC7887378 33589525

[6] Cinier J Hubert M Besson L Di Roio A Rodriguez C Lombardi V. Recruitment and expansion of tregs cells in the tumor environment-how to target them? Cancers (Basel). (2021) 13. doi: 10.3390/cancers13081850 PMC8069615 33924428

[7] Hosseinalizadeh H Rabiee F Eghbalifard N Rajabi H Klionsky DJ Rezaee A. Regulating the regulatory T cells as cell therapies in autoimmunity and cancer. Front Med (Lausanne). (2023) 10:1244298. doi: 10.3389/fmed.2023.1244298 37828948 PMC10565010

[8] Tanaka A Sakaguchi S. Regulatory T cells in cancer immunotherapy. Cell Res. (2017) 27:109–18. doi: 10.1038/cr.2016.151 PMC5223231 27995907

[9] Chaudhary B Elkord E. Regulatory T cells in the tumor microenvironment and cancer progression: role and therapeutic targeting. Vaccines (Basel). (2016) 4. doi: 10.3390/vaccines4030028 PMC5041022 27509527

[10] McRitchie BR Akkaya B. Exhaust the exhausters: Targeting regulatory T cells in the tumor microenvironment. Front Immunol. (2022) 13:940052. doi: 10.3389/fimmu.2022.940052 36248808 PMC9562032

[11] Scott EN Gocher AM Workman CJ Vignali DAA. Regulatory T cells: barriers of immune infiltration into the tumor microenvironment. Front Immunol. (2021) 12:702726. doi: 10.3389/fimmu.2021.702726 34177968 PMC8222776

[12] Konig M Rharbaoui F Aigner S Dalken B Schuttrumpf J. Tregalizumab - A monoclonal antibody to target regulatory T cells. Front Immunol. (2016) 7:11. doi: 10.3389/fimmu.2016.00011 26834751 PMC4724712

[13] Sampson JH Schmittling RJ Archer GE Congdon KL Nair SK Reap EA. A pilot study of IL-2Ralpha blockade during lymphopenia depletes regulatory T-cells and correlates with enhanced immunity in patients with glioblastoma. PloS One. (2012) 7:e31046. doi: 10.1371/journal.pone.0031046 22383993 PMC3288003

[14] Dranoff G. CTLA-4 blockade: unveiling immune regulation. J Clin Oncol. (2005) 23:662–4. doi: 10.1200/JCO.2005.09.923 15613692

[15] Scurr M Pembroke T Bloom A Roberts D Thomson A Smart K. Low-dose cyclophosphamide induces antitumor T-cell responses, which associate with survival in metastatic colorectal cancer. Clin Cancer Res. (2017) 23:6771–80. doi: 10.1158/1078-0432.CCR-17-0895 PMC5769815 28855352

[16] Galluzzi L Senovilla L Zitvogel L Kroemer G. The secret ally: immunostimulation by anticancer drugs. Nat Rev Drug Discov. (2012) 11:215–33. doi: 10.1038/nrd3626 22301798

[17] Hobeika AC Morse MA Osada T Peplinski S Lyerly HK Clay TM. Depletion of human regulatory T cells. Methods Mol Biol. (2011) 707:219–31. doi: 10.1007/978-1-61737-979-6_14 21287338

[18] Zou W. Regulatory T cells, tumour immunity and immunotherapy. Nat Rev Immunol. (2006) 6:295–307. doi: 10.1038/nri1806 16557261

[19] Liu C Workman CJ Vignali DA. Targeting regulatory T cells in tumors. FEBS J. (2016) 283:2731–48. doi: 10.1111/febs.13656 26787424

[20] Toor SM Syed Khaja AS Alkurd I Elkord E. *In-vitro* effect of pembrolizumab on different T regulatory cell subsets. Clin Exp Immunol. (2018) 191:189–97. doi: 10.1111/cei.13060 PMC5758372 28963773

[21] Jacobs JF Punt CJ Lesterhuis WJ Sutmuller RP Brouwer HM Scharenborg NM. Dendritic cell vaccination in combination with anti-CD25 monoclonal antibody treatment: a phase I/II study in metastatic melanoma patients. Clin Cancer Res. (2010) 16:5067–78. doi: 10.1158/1078-0432.CCR-10-1757 20736326

[22] Yamada Y Aoyama A Tocco G Boskovic S Nadazdin O Alessandrini A. Differential effects of denileukin diftitox IL-2 immunotoxin on NK and regulatory T cells in nonhuman primates. J Immunol. (2012) 188:6063–70. doi: 10.4049/jimmunol.1200656 PMC3370077 22586034

[23] Attia P Maker AV Haworth LR Rogers-Freezer L Rosenberg SA. Inability of a fusion protein of IL-2 and diphtheria toxin (Denileukin Diftitox, DAB389IL-2, ONTAK) to eliminate regulatory T lymphocytes in patients with melanoma. J Immunother. (2005) 28:582–92. doi: 10.1097/01.cji.0000175468.19742.10 PMC1533764 16224276

[24] Powell DJ Jr. Felipe-Silva A Merino MJ Ahmadzadeh M Allen T Levy C. Administration of a CD25-directed immunotoxin, LMB-2, to patients with metastatic melanoma induces a selective partial reduction in regulatory T cells in vivo. J Immunol. (2007) 179:4919–28. doi: 10.4049/jimmunol.179.7.4919 PMC2134981 17878392

[25] Ge Y Domschke C Stoiber N Schott S Heil J Rom J. Metronomic cyclophosphamide treatment in metastasized breast cancer patients: immunological effects and clinical outcome. Cancer immunol immunother: CII. (2012) 61:353–62. doi: 10.1007/s00262-011-1106-3 PMC11028651 21915801

[26] von Boehmer H Daniel C. Therapeutic opportunities for manipulating T(Reg) cells in autoimmunity and cancer. Nat Rev Drug Discov. (2013) 12:51–63. doi: 10.1038/nrd3683 23274471

[27] Munn DH Sharma MD Johnson TS. Treg destabilization and reprogramming: implications for cancer immunotherapy. Cancer Res. (2018) 78:5191–9. doi: 10.1158/0008-5472.CAN-18-1351 PMC6139039 30181177

[28] Golgher D Jones E Powrie F Elliott T Gallimore A. Depletion of CD25+ regulatory cells uncovers immune responses to shared murine tumor rejection antigens. Eur J Immunol. (2002) 32:3267–75. doi: 10.1002/1521-4141(200211)32:11<3267::AID-IMMU3267>3.0.CO;2-1 12555672

[29] Jones E Dahm-Vicker M Simon AK Green A Powrie F Cerundolo V. Depletion of CD25+ regulatory cells results in suppression of melanoma growth and induction of autoreactivity in mice. Cancer Immun. (2002) 2:1. 12747746

[30] Akimova T Zhang T Negorev D Singhal S Stadanlick J Rao A. Human lung tumor FOXP3+ Tregs upregulate four “Treg-locking” transcription factors. JCI Insight. (2017) 2:94075. doi: 10.1172/jci.insight.94075 28814673 PMC5621877

[31] Lal G Bromberg JS. Epigenetic mechanisms of regulation of Foxp3 expression. Blood. (2009) 114:3727–35. doi: 10.1182/blood-2009-05-219584 PMC2773485 19641188

[32] Fortin M D’Anjou H Higgins ME Gougeon J Aube P Moktefi K. A multi-target antisense approach against PDE4 and PDE7 reduces smoke-induced lung inflammation in mice. Respir Res. (2009) 10:39. doi: 10.1186/1465-9921-10-39 19457265 PMC2696437

[33] Guimond A Viau E Aube P Renzi PM Paquet L Ferrari N. Advantageous toxicity profile of inhaled antisense oligonucleotides following chronic dosing in non-human primates. Pulm Pharmacol Ther. (2008) 21:845–54. doi: 10.1016/j.pupt.2008.08.001 18761414

[34] Souleimanian N Deleavey GF Soifer H Wang S Tiemann K Damha MJ. Antisense 2’-deoxy, 2’-fluroarabino nucleic acids (2’F-ANAs) oligonucleotides: *in vitro* gymnotic silencers of gene expression whose potency is enhanced by fatty acids. Mol Ther Nucleic Acids. (2012) 1:e43. doi: 10.1038/mtna.2012.35 23344235 PMC3499694

[35] Smaldone G Beneduce G Incoronato M Pane K Franzese M Coppola L. KCTD15 is overexpressed in human childhood B-cell acute lymphoid leukemia. Sci Rep. (2019) 9:20108. doi: 10.1038/s41598-019-56701-7 31882877 PMC6934626

[36] Chorzalska A Morgan J Ahsan N Treaba DO Olszewski AJ Petersen M. Bone marrow-specific loss of ABI1 induces myeloproliferative neoplasm with features resembling human myelofibrosis. Blood. (2018) 132:2053–66. doi: 10.1182/blood-2018-05-848408 PMC6236464 30213875

[37] Hensel KO Cantner F Bangert F Wirth S Postberg J. Episomal HBV persistence within transcribed host nuclear chromatin compartments involves HBx. Epigenet Chromatin. (2018) 11:34. doi: 10.1186/s13072-018-0204-2 PMC6015472 29933745

[38] Frank S Ahuja G Bartsch D Russ N Yao W Kuo JC. yylncT defines a class of divergently transcribed lncRNAs and safeguards the T-mediated mesodermal commitment of human PSCs. Cell Stem Cell. (2019) 24:318–27 e8. doi: 10.1016/j.stem.2018.11.005 30554961

[39] Takahashi M Li H Zhou J Chomchan P Aishwarya V Damha MJ. Dual mechanisms of action of self-delivering, anti-HIV-1 FANA oligonucleotides as a potential new approach to HIV therapy. Mol Ther Nucleic Acids. (2019) 17:615–25. doi: 10.1016/j.omtn.2019.07.001 PMC6695270 31394430

[40] Ferrari N Bergeron D Tedeschi AL Mangos MM Paquet L Renzi PM. Characterization of antisense oligonucleotides comprising 2’-deoxy-2’-fluoro-beta-D-arabinonucleic acid (FANA): specificity, potency, and duration of activity. Ann N Y Acad Sci. (2006) 1082:91–102. doi: 10.1196/annals.1348.032 17145930

[41] Karaki S Paris C Rocchi P. Antisense oligonucleotides, a novel developing targeting therapy. Antisense Ther. (2019) 10. doi: 10.5772/intechopen.82105

[42] Khvorova A Watts JK. The chemical evolution of oligonucleotide therapies of clinical utility. Nat Biotechnol. (2017) 35:238–48. doi: 10.1038/nbt.3765 PMC5517098 28244990

[43] Martin-Pintado N Yahyaee-Anzahaee M Campos-Olivas R Noronha AM Wilds CJ Damha MJ. The solution structure of double helical arabino nucleic acids (ANA and 2’F-ANA): effect of arabinoses in duplex-hairpin interconversion. Nucleic Acids Res. (2012) 40:9329–39. doi: 10.1093/nar/gks672 PMC3467067 22798499

[44] Amanat M Nemeth CL Fine AS Leung DG Fatemi A. Antisense oligonucleotide therapy for the nervous system: from bench to bedside with emphasis on pediatric neurology. Pharmaceutics. (2022) 14. doi: 10.3390/pharmaceutics14112389 PMC9695718 36365206

[45] Akimova T Levine MH Beier UH Hancock WW. Standardization, evaluation, and area-under-curve analysis of human and murine Treg suppressive function. Methods Mol Biol. (2016) 1371:43–78. doi: 10.1007/978-1-4939-3139-2_4 26530794 PMC5554116

[46] Jackaman C Bundell CS Kinnear BF Smith AM Filion P van Hagen D. IL-2 intratumoral immunotherapy enhances CD8+ T cells that mediate destruction of tumor cells and tumor-associated vasculature: a novel mechanism for IL-2. J Immunol. (2003) 171:5051–63. doi: 10.4049/jimmunol.171.10.5051 14607902

[47] Livak KJ Schmittgen TD. Analysis of relative gene expression data using real-time quantitative PCR and the 2(-Delta Delta C(T)) Method. Methods. (2001) 25:402–8. doi: 10.1006/meth.2001.1262 11846609

[48] Liu Y Wang L Han R Beier UH Hancock WW. Two lysines in the forkhead domain of foxp3 are key to T regulatory cell function. PloS One. (2012) 7:e29035. doi: 10.1371/journal.pone.0029035 22247766 PMC3256141

[49] Ruffo E Wu RC Bruno TC Workman CJ Vignali DAA. Lymphocyte-activation gene 3 (LAG3): The next immune checkpoint receptor. Semin Immunol. (2019) 42:101305. doi: 10.1016/j.smim.2019.101305 31604537 PMC6920665

[50] Hossen MM Ma Y Yin Z Xia Y Du J Huang JY. Current understanding of CTLA-4: from mechanism to autoimmune diseases. Front Immunol. (2023) 14:1198365. doi: 10.3389/fimmu.2023.1198365 37497212 PMC10367421

[51] Avery L Filderman J Szymczak-Workman AL Kane LP. Tim-3 co-stimulation promotes short-lived effector T cells, restricts memory precursors, and is dispensable for T cell exhaustion. Proc Natl Acad Sci U S A. (2018) 115:2455–60. doi: 10.1073/pnas.1712107115 PMC5877951 29463725

[52] Chauvin JM Zarour HM. TIGIT in cancer immunotherapy. J Immunother Cancer. (2020) 8. doi: 10.1136/jitc-2020-000957 PMC7477968 32900861

[53] Simon S Labarriere N. PD-1 expression on tumor-specific T cells: Friend or foe for immunotherapy? Oncoimmunology. (2017) 7:e1364828. doi: 10.1080/2162402X.2017.1364828 29296515 PMC5739549

[54] Dees S Ganesan R Singh S Grewal IS. Regulatory T cell targeting in cancer: Emerging strategies in immunotherapy. Eur J Immunol. (2021) 51:280–91. doi: 10.1002/eji.202048992 33302322

[55] Shan F Somasundaram A Bruno TC Workman CJ Vignali DAA. Therapeutic targeting of regulatory T cells in cancer. Trends Cancer. (2022) 8:944–61. doi: 10.1016/j.trecan.2022.06.008 PMC9588644 35853825

[56] Tanaka A Sakaguchi S. Targeting Treg cells in cancer immunotherapy. Eur J Immunol. (2019) 49:1140–6. doi: 10.1002/eji.201847659 31257581

[57] Revenko A Carnevalli LS Sinclair C Johnson B Peter A Taylor M. Direct targeting of FOXP3 in Tregs with AZD8701, a novel antisense oligonucleotide to relieve immunosuppression in cancer. J Immunother Cancer. (2022) 10. doi: 10.1136/jitc-2021-003892 PMC8987763 35387780

[58] Denisov AY Noronha AM Wilds CJ Trempe JF Pon RT Gehring K. Solution structure of an arabinonucleic acid (ANA)/RNA duplex in a chimeric hairpin: comparison with 2’-fluoro-ANA/RNA and DNA/RNA hybrids. Nucleic Acids Res. (2001) 29:4284–93. doi: 10.1093/nar/29.21.4284 PMC60200 11691916

[59] Lok CN Viazovkina E Min KL Nagy E Wilds CJ Damha MJ. Potent gene-specific inhibitory properties of mixed-backbone antisense oligonucleotides comprised of 2’-deoxy-2’-fluoro-D-arabinose and 2’-deoxyribose nucleotides. Biochemistry. (2002) 41:3457–67. doi: 10.1021/bi0115075 11876654

[60] Min KL Viazovkina E Galarneau A Parniak MA Damha MJ. Oligonucleotides comprised of alternating 2’-deoxy-2’-fluoro-beta-D-arabinonucleosides and D-2’-deoxyribonucleosides (2’F-ANA/DNA ‘altimers’) induce efficient RNA cleavage mediated by RNase H. Bioorg Med Chem Lett. (2002) 12:2651–4. doi: 10.1016/S0960-894X(02)00439-0 12182880

[61] Moroz E Lee SH Yamada K Halloy F Martinez-Montero S Jahns H. Carrier-free gene silencing by amphiphilic nucleic acid conjugates in differentiated intestinal cells. Mol Ther Nucleic Acids. (2016) 5:e364. doi: 10.1038/mtna.2016.69 27648924 PMC5056993

[62] Kalota A Karabon L Swider CR Viazovkina E Elzagheid M Damha MJ. 2’-deoxy-2’-fluoro-beta-D-arabinonucleic acid (2’F-ANA) modified oligonucleotides (ON) effect highly efficient, and persistent, gene silencing. Nucleic Acids Res. (2006) 34:451–61. doi: 10.1093/nar/gkj455 PMC1342038 16421272

[63] Liang XH Sun H Nichols JG Crooke ST. RNase H1-dependent antisense oligonucleotides are robustly active in directing RNA cleavage in both the cytoplasm and the nucleus. Mol Ther. (2017) 25:2075–92. doi: 10.1016/j.ymthe.2017.06.002 PMC5589097 28663102

[64] Della Valle F Reddy P Yamamoto M Liu P Saera-Vila A Bensaddek D. LINE-1 RNA causes heterochromatin erosion and is a target for amelioration of senescent phenotypes in progeroid syndromes. Sci Transl Med. (2022) 14:eabl6057. doi: 10.1126/scitranslmed.abl6057 35947677

[65] Pelisch N Rosas Almanza J Stehlik KE Aperi BV Kroner A. Use of a self-delivering anti-CCL3 FANA oligonucleotide as an innovative approach to target inflammation after spinal cord injury. eNeuro. (2021) 8. doi: 10.1523/ENEURO.0338-20.2021 PMC7986543 33632814

[66] Russell BL Sooklal SA Malindisa ST Daka LJ Ntwasa M. The tumor microenvironment factors that promote resistance to immune checkpoint blockade therapy. Front Oncol. (2021) 11:641428. doi: 10.3389/fonc.2021.641428 34268109 PMC8276693

[67] Schoenfeld AJ Hellmann MD. Acquired resistance to immune checkpoint inhibitors. Cancer Cell. (2020) 37:443–55. doi: 10.1016/j.ccell.2020.03.017 PMC7182070 32289269

[68] de Britto Evangelista GF Figueiredo AB de Barros ESMJ Gollob KJ. Balancing the good and the bad: controlling immune-related adverse events versus anti-tumor responses in cancer patients treated with immune checkpoint inhibitors. Immunother Adv. (2022) 2:ltac008. doi: 10.1093/immadv/ltac008 35919497 PMC9327097

[69] Barrueto L Caminero F Cash L Makris C Lamichhane P Deshmukh RR. Resistance to checkpoint inhibition in cancer immunotherapy. Transl Oncol. (2020) 13:100738. doi: 10.1016/j.tranon.2019.12.010 32114384 PMC7047187

[70] Sakuishi K Ngiow SF Sullivan JM Teng MW Kuchroo VK Smyth MJ. TIM3(+)FOXP3(+) regulatory T cells are tissue-specific promoters of T-cell dysfunction in cancer. Oncoimmunology. (2013) 2:e23849. doi: 10.4161/onci.23849 23734331 PMC3654601

[71] Saleh R Elkord E. Treg-mediated acquired resistance to immune checkpoint inhibitors. Cancer letters. (2019) 457:168–79. doi: 10.1016/j.canlet.2019.05.003 31078738

