# Supplementary material for: Antisense targeting of FOXP3+ Tregs to boost anti-tumor immunity
Source: Front Immunol. 2024 Aug 21;15:1426657. doi: 10.3389/fimmu.2024.1426657 (PMC11371716; doi:10.3389/fimmu.2024.1426657)
Supplement: Supplementary file 1 [file DataSheet1.docx]

Supplementary Material

**Antisense targeting of FOXP3+ Tregs to boost anti-tumor immunity**

**Tatiana Akimova, Liqing Wang, Zhanna Bartosh, Lanette M. Christensen, Evgeniy Eruslanov, Sunil Singhal, Veenu Aishwarya and Wayne W Hancock***

* Correspondence: Wayne W Hancock: whancock@pennmedicine.upenn.edu

# Supplementary materials and methods

*Cell culture and cell preparation reagents.* The enzymatic cocktail for tumor digestion consisted of serum-free Hyclone Leibovitz L-15 medium, supplemented with 1% penicillin-streptomycin, collagenase type I and IV (170 mg/L = 45-60 U/mL), collagenase type II (56 mg/L = 15-20 U/mL), DNase I (25 mg/L) and elastase (25 mg/L), all from Worthington Biochemical. T cell media consisted with RPMI 1640 (Invitrogen), supplemented with 10% heat-inactivated FBS, penicillin and streptomycin, and 2-mercaptoethanol (100 μM). T cell media was used for human Treg suppression assay, for screening experiments with PBMC (evaluation of ASO toxicity) and for the experiments of PBMC and ASO treatment of isolated human Tregs. For cytokines expression assays, evaluated by flow cytometry, we used HBSS media, supplemented with 10% heat-inactivated FBS. For other experiments with ASO or Scramble treated PBMC or cancer samples, we used DMEM, supplemented with 3% of FBS, with an addition of NH_4_Cl 3mM and Arsenic 1μM to enhance gymnosis, as reported (1).

*Flow cytometry* was performed with additional controls for cryopreservation and fixation/permeabilization artefacts. To do so, we have included splenocytes and LN cells from WT B6 tumor-free healthy mice into each murine panel, and included the healthy donors PBMC controls into human flow cytometry panels. Each new marker (antibodies for checkpoint molecules) was evaluated serially on non-fixed, and then on fixed samples to ensure a reasonable performance. Cryopreservation artifacts were controlled by comparison of data obtained from 2 aliquots of the same sample, before and after cryopreservation. We used live/dead (or Ghost or Zombie, Table S1) fixable reagent, then washed cells, applied FC blocking reagents (Human TruStain FcX or murine CD16/32) and monocytes blocking reagent (Table S1) for 5-10 min at room temperature, then stained for surface markers for 40 min, 4°C in pre-titrated concentrations, then washed, and performed Fixation/Permeabilization step or stimulate cells.

In experiments with cytokines expression, cells were stimulated for 4 hours with phorbol myristate acetate (PMA, 3 ng/ml) and ionomycin (1 µM) + Monensin (Biolegend), then fixed with BD Cytofix/Cytoperm™ Fixation/Permeabilization Solution kit, according to manufacturer’s instruction, and then stained with pre-titrated antibodies against corresponding cytokines for 40-50 min, 4°C. In experiments with FOXP3 staining, after superficial markers staining step, cells were fixed and then permeabilized using Transcription Factor Buffer Set according to manufacturer’s instruction, and then stained with corresponding antibodies for 1 h 4°C. We used single color stained samples and FMO control samples to ensure proper gating strategy.

*RT-qPCR.* After RNA isolation, we evaluated RNA quality and concentration using Nanodrop 2000. Then we synthesized cDNA and either run TaqMan gene expression assay or performed cDNA amplification using TaqMan® PreAmp Master Mix Kit, cat#4384267, according to manufacturer’s instruction, with 14 cycles of pre-amplification. The decision of pre-amplification was made when we had a low concentration of RNA (isolated from low number of cells or from the small tumors), and when we planned to test expression of multiple genes. All samples of the same type from the same experiment, i.e. Scramble controls vs. all ASO treated samples, had the same method of processing, i.e. all of them were either pre-amplified or were not. In most experiments, RNA concentration allowed us to compare FOXP3 gene expression in two separate experiments: with pre-amplification and without pre-amplification, to ensure an absence of possible artefacts due to cDNA pre-amplification. All primers used for qPCR (ThermoFisher Scientific) are listed in Supplementary Table S1.

*PCA analysis.* We used Z-scored flow cytometry data. Variables include Treg numbers in CD4+ subset, expression of exhaustion markers in different subsets of cells, and production of inflammatory cytokines by those cells. Due to restricted amount of intratumoral cells, we were unable to test all cytokines in all available samples, and had to exclude from the PCA analysis data of IL-10, TNFα, IL-6 and Perforin expression. The assumption of independent sampling was met. The assumptions of normality, linear relationships between pairs of variables, and the variables being correlated at a moderate level were checked and met. Two components were extracted, based on the eigenvalues over 1 criterion and the scree plot. Direct Oblimin rotation was applied. More details are reported in Supplementary Table S2.

#
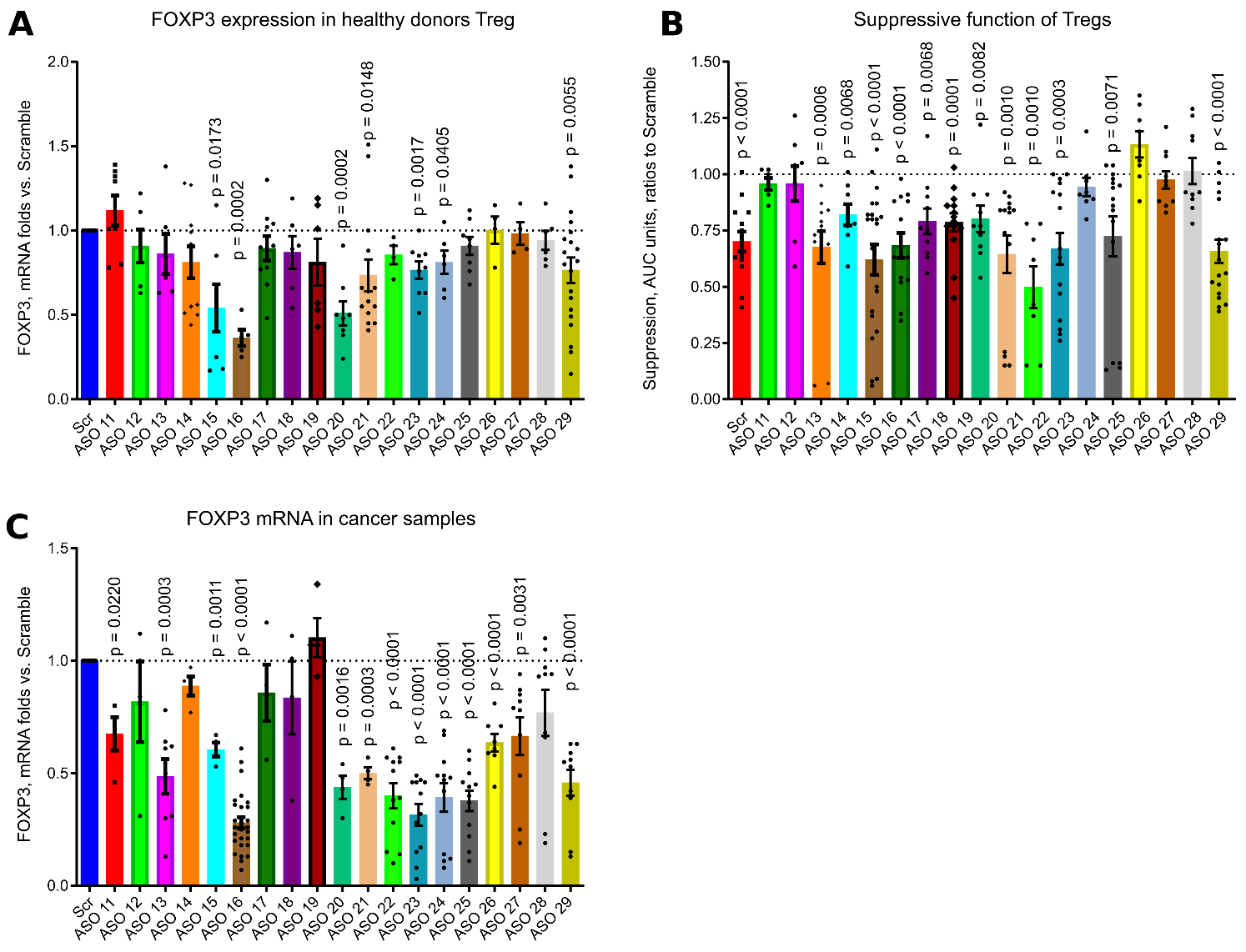
Supplementary Figures

**Fig****ure S1. A,** healthy donor Tregs, isolated from 9 donors in 9 experiments were stimulated with CD3/28 microbeads, 1.3 beads/cell, in presence of 1.5 μM of Scramble or ASOs FOXP3 for overnight, then evaluated by qPCR. **B,** healthy donor Tregs, isolated from 13 donors in 13 experiments were incubated with 2.5 μM of Scramble of ASO FOXP3 for 3.5 hours, then washed twice and used in 5-6 days suppression assay with autologous or allogeneic responders from 10 donors. **C**, 3 lung cancer and 1 melanoma tumor samples, 6 pleural effusion samples, 4 lung cancer distant lung and 2 lymph nodes from 11 patients with lung cancer in 7 experiments, were stimulated with CD3/28 microbeads, 0.3 beads/cell and treated with 1.5μM of Scramble or ASOs for 5 days, then evaluated by qPCR.

**A-C**, one sample T-tests with mean =1, p values are shown for the results with p<0.05.

**
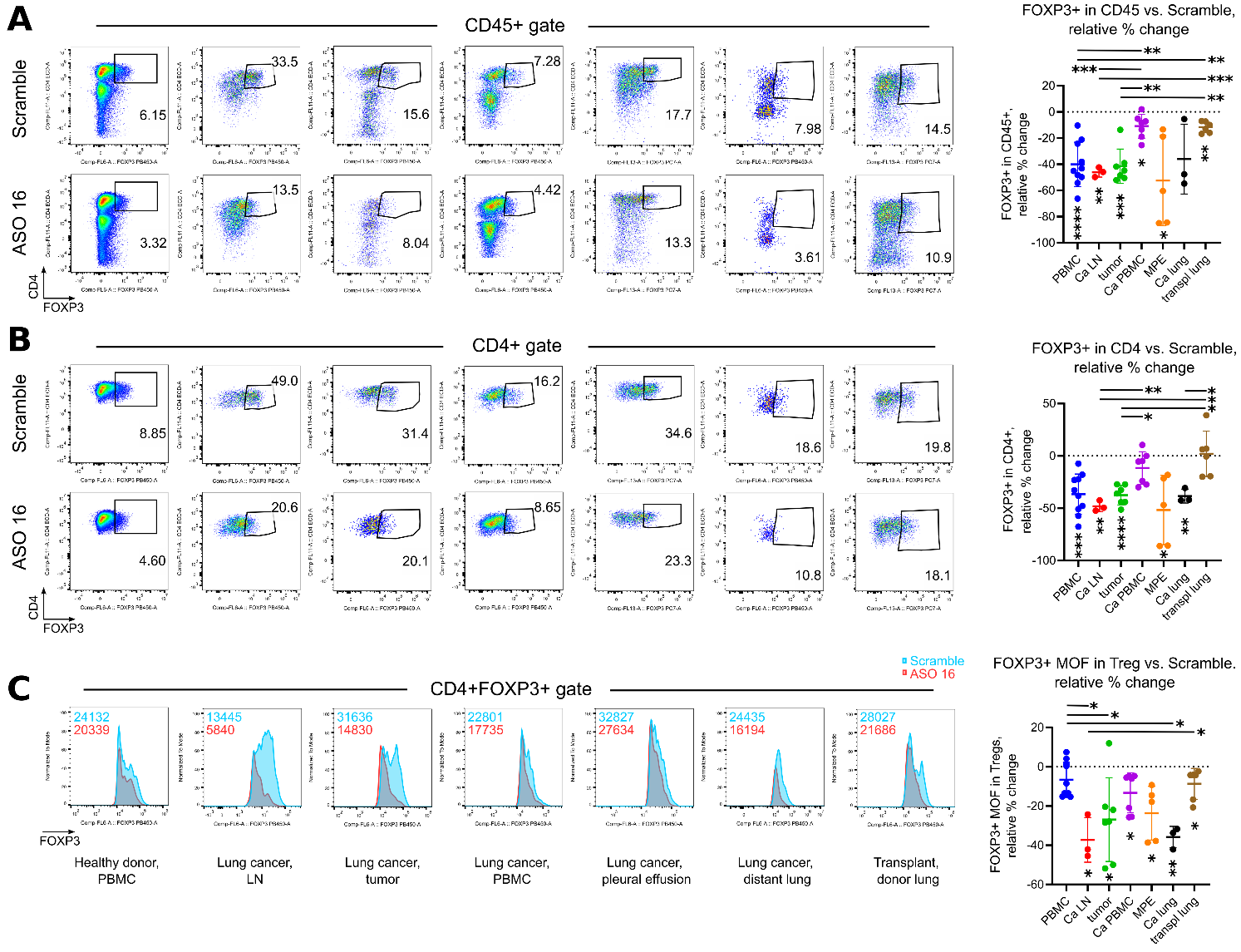
**

**Figure S2. A-C**, multiple cancer, non-cancer and healthy donors PBMC samples were evaluated in five flow cytometry experiments with 2-3 different panels for each experiment, as in Figure 3F. Cells were stimulated with CD3/28 microbeads, 0.3 beads/cell and treated with 1.5μM of Scramble or ASO 16 for 5 days. Samples consisted of: 5 healthy donors PBMC, 3 lung cancer tumor samples and 1 mesothelioma tumor sample, 4 tumor-free lung cancer samples received during a surgery, 3 lung cancer pleural effusion samples, 3 lung draining lymph nodes cancer samples and 4 lung cancer PBMC samples from 10 lung cancer and 1 mesothelioma patients. We also evaluated 3 transplant lungs, rejected for transplant, and 6 Treg-depleted samples. Treg -depleted samples consisted of: 2 healthy donors PBMC, 1 lung cancer LN, 2 lung cancer tumor samples and 1 pleural effusion sample. **A-B**, representative flow cytometry plots with % of Tregs in (**A**) viable CD45+ subset and (**B**) in CD4+ gated T cells with corresponding statistics on the right. **C**, representative histograms (left) with corresponding statistics (right) of FOXP3 expression in leftover Tregs that still expressed FOXP3 in the end of treatment. Median of fluorescence (MOF) values are shown in the left corners.

**A-C**, one sample T-tests with mean = 0, only results with p<0.05 are shown using asterisks below samples. **A-C**, Brown-Forsythe and Welch one way ANOVA tests with Games-Howell's multiple comparisons test. Results are shown by horizontal lines with asterisks above samples. * p<0.05, ** p<0.01, and ***p<0.001

**
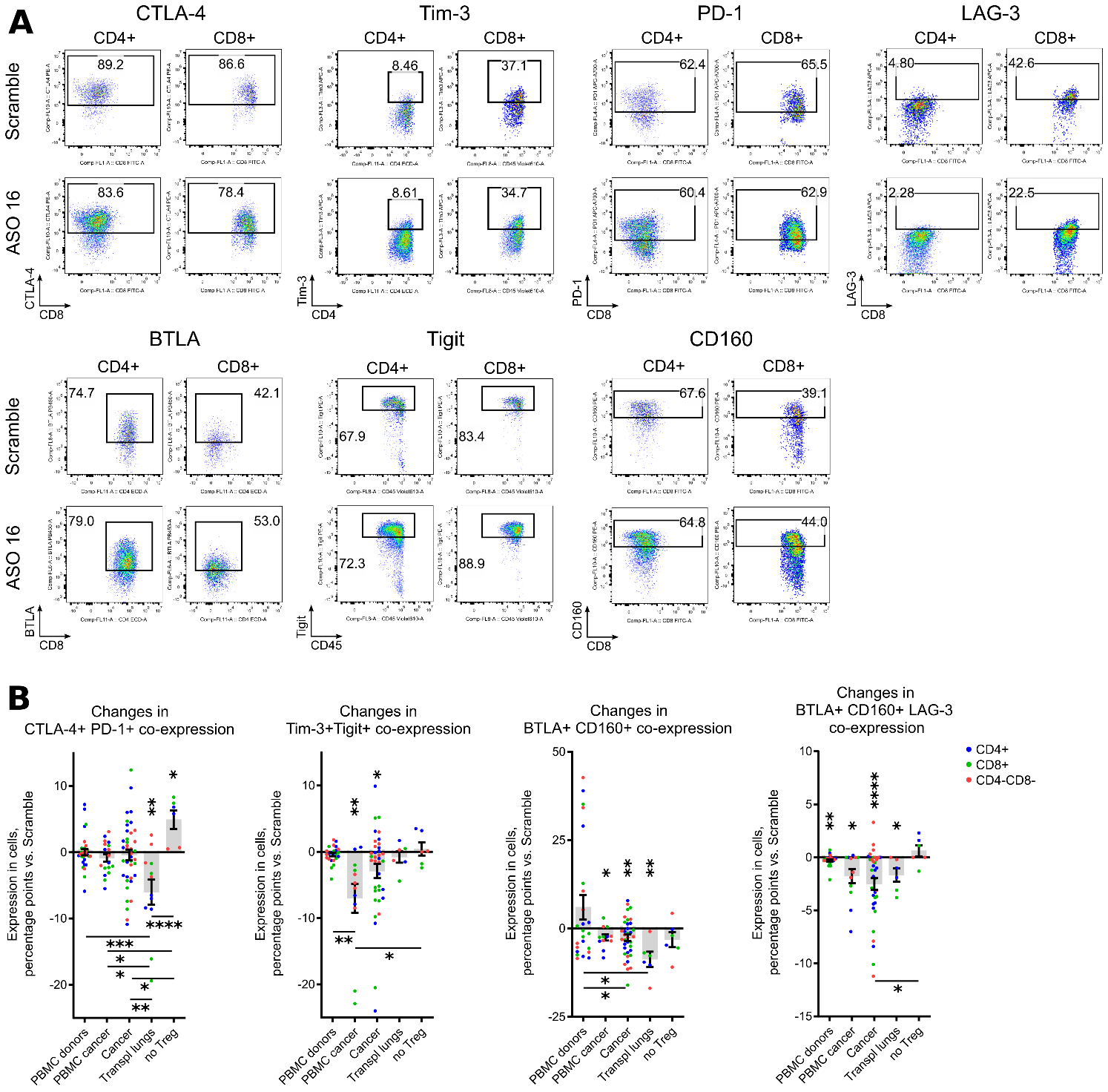
Figure S3. A-B,** Same samples as in Supplementary Figure S2. **A**, representative flow cytometry plots showing expression of exhaustion markers in tumor samples. **B**, cells were evaluated for co-expression of two and more exhaustion markers.

**B,** except for BTLA+CD160+LAG-3+ co expression - one sample T-tests with mean = 0, only results with p<0.05 are shown using asterisks above samples. **B**, except for BTLA+CD160+LAG-3+ co expression - one-way ANOVA with Tukey’s multiple comparison tests, results are shown by horizontal lines with asterisks below samples. **B**, BTLA+CD160+LAG-3+ - one sample Wilcoxon rank tests with median = 0, only results with p<0.05 are shown using asterisks above samples. **B**, BTLA+CD160+LAG-3+ - Kruskal-Wallis test with Dunn’s multiple comparison tests, results are shown by horizontal lines with asterisks below samples. * p<0.05, ** p<0.01, ***p<0.001 and **** p<0.0001


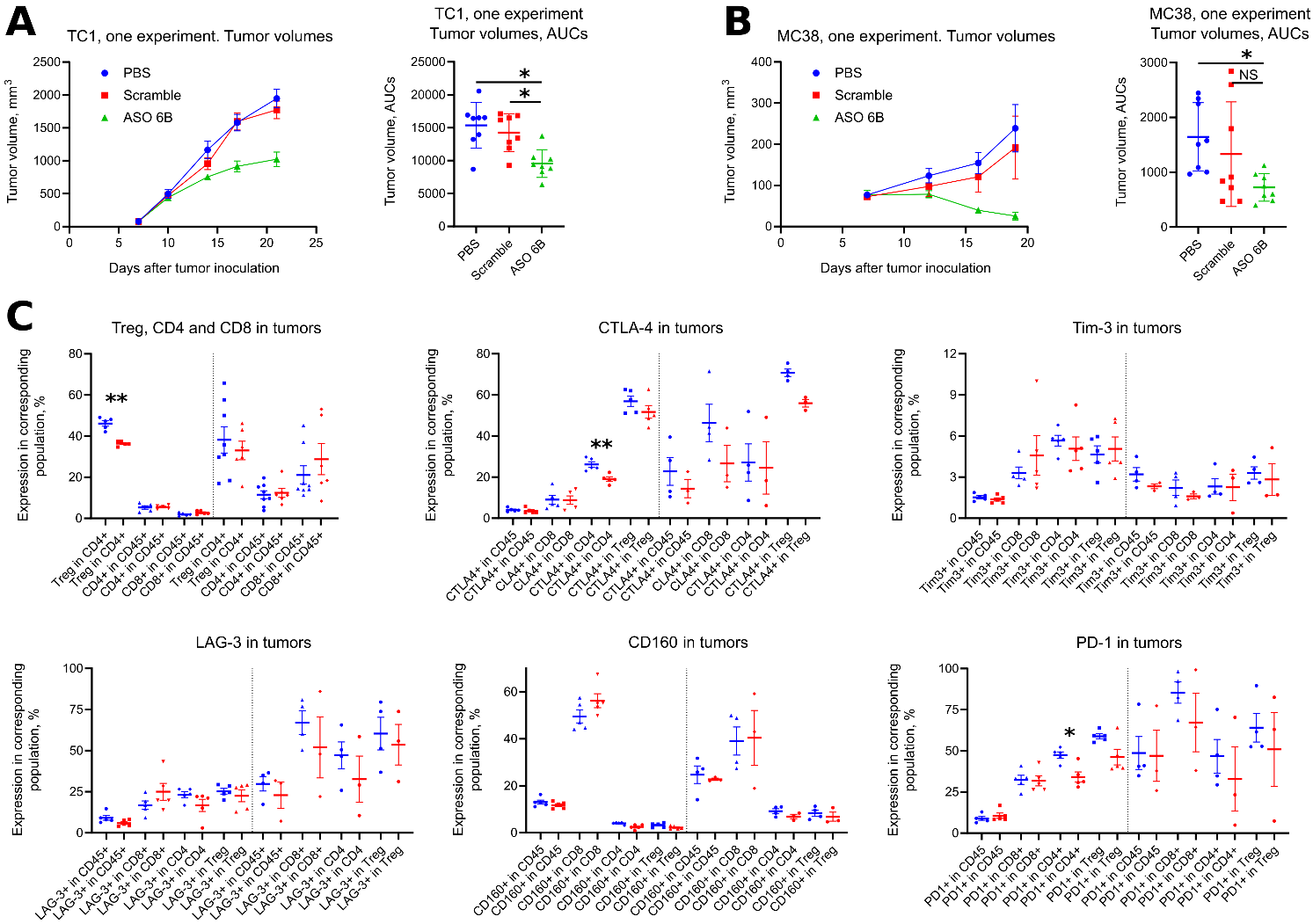


**Figure S4.** Mice in two tumor models, TC1 and MC38 were treated as in Figure 4 E, F. **A-B**, non-normalized data of tumor growth (left) and corresponding area under curves of tumor growths were calculated and compared for (**A**) 8 mice per group in one TC1 experiment and (**B**) 8 mice per group in one MC38 experiment. **C**, 5 TC1 tumors from one experiment and 8 MC38 tumors from one experiment were harvested at day 14^th^ after tumor inoculation and evaluated by flow cytometry. MC38 tumors, due to the small size, especially in ASO treated groups, were combined together as 2-3 tumors per one flow cytometry sample. For Treg, CD4 and CD8 data, results from two different flow cytometry panels are combined in the group of MC38 mice. TC1 mice are shown on the left of each graph, and MC38 mice are shown on the right of each graph.

**A-B**, Kruskal-Wallis test with Dunn’s multiple comparison tests. **C**, Mann-Whitney tests, only results with p<0.05 are shown using asterisks above samples. * p<0.05, ** p<0.01, NS – not significant

**
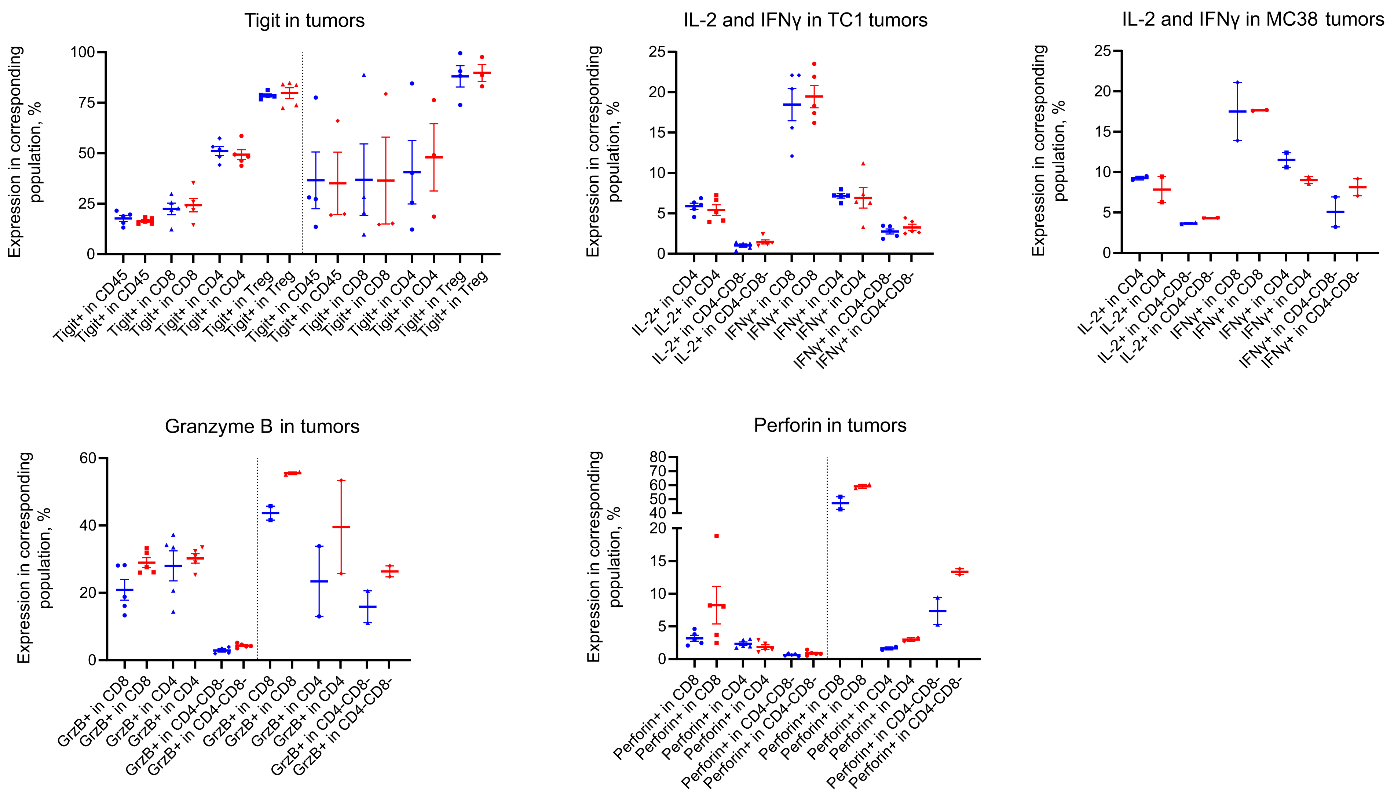
**

**Figure S5.** Same mice as in Supplementary Figure S4C. Tumor samples were stimulated for 4 h with PMA/ionomycin with Monensin, then evaluated for cytokines expression. Tigit expression was evaluated in non-stimulated cells, as in S4C. Data from one TC1 (on the left in each graph) and one MC38 experiment (on the right in each graph) are shown.


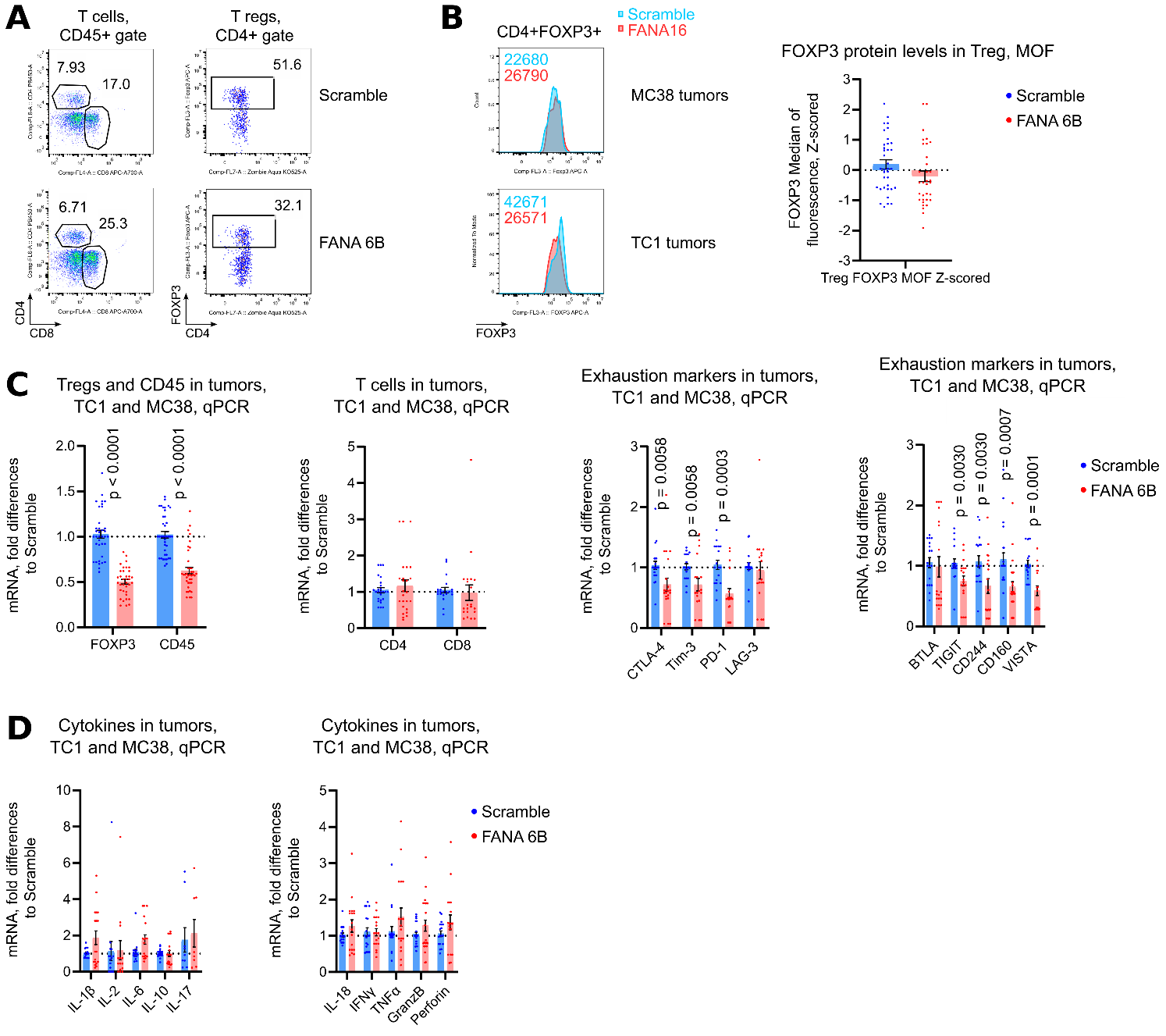


**Figure S6. A-B,** 26 TC1 tumors and 35 MC38 tumors were harvested at day 14^th^ after tumor inoculation. MC38 tumors, due to the small size, especially in ASO treated groups, were combined together as 2-3 tumors per one flow cytometry sample. **A**, representative plots of T cells (left) and Treg (right) staining in MC38 tumors. **B**, representative histograms of FOXP3 MOF in intratumoral CD4+FOXP3+ Tregs, MC38 tumors (top) and TC1 tumors (bottom) and **C**, corresponding statistics with Z-scored flow cytometry data. **C-D,** 18 TC1 tumors and 16 MC38 tumors were harvested at day 14^th^ after tumor inoculation and evaluated by qPCR. MC38 tumors, due to the small size, especially in ASO treated groups, were combined together as 2-3 tumors per one RNA sample.

**C-D,** multiple T-tests FDR Benjamini, Krieger and Yekutieli, with Q = 5%, only results with p<0.05 are shown.


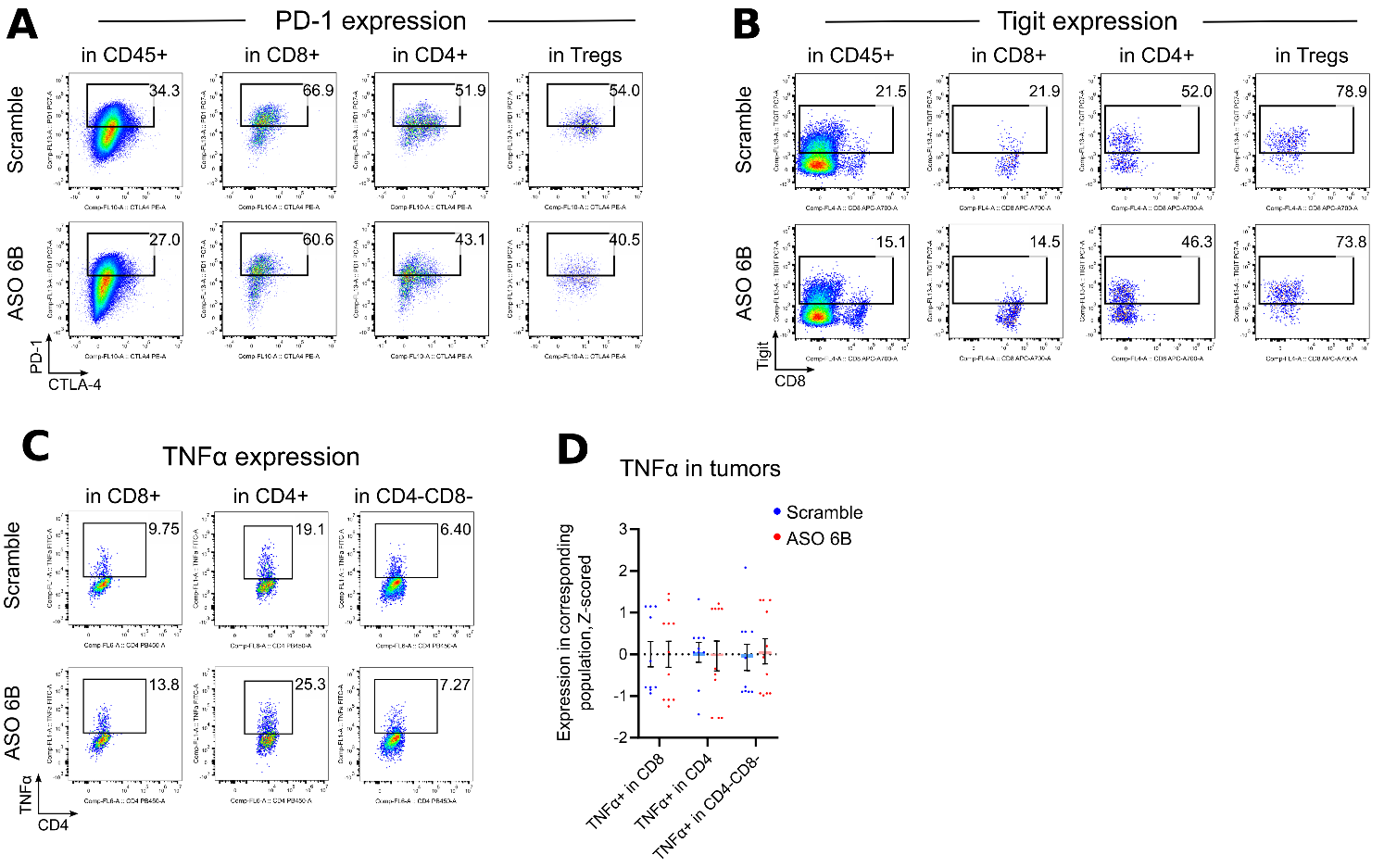
**Figure S7. A-D,** 26 TC1 tumors and 35 MC38 tumors were harvested at day 14^th^ after tumor inoculation. MC38 tumors, due to the small size, especially in ASO treated groups, were combined together as 2-3 tumors per one flow cytometry sample. **A-B**, representative flow cytometry plots of (**A**) PD-1 and (**B**) Tigit expression in TC1 tumors are shown. Corresponding statistics are shown in Figure 5G. **C-D**, 19 TC1 and 12 MC38 tumors as in Figure 5 B-C and E-G, were stimulated for 4 h with PMA/ionomycin with Monensin, then evaluated for cytokines expression. **C**, Flow cytometry plots show representative data with staining for TNFα with corresponding statistics (**D**) are shown.

**D**, multiple T-tests FDR Benjamini, Krieger and Yekutieli, with Q = 5%. There were no significant differences.

**
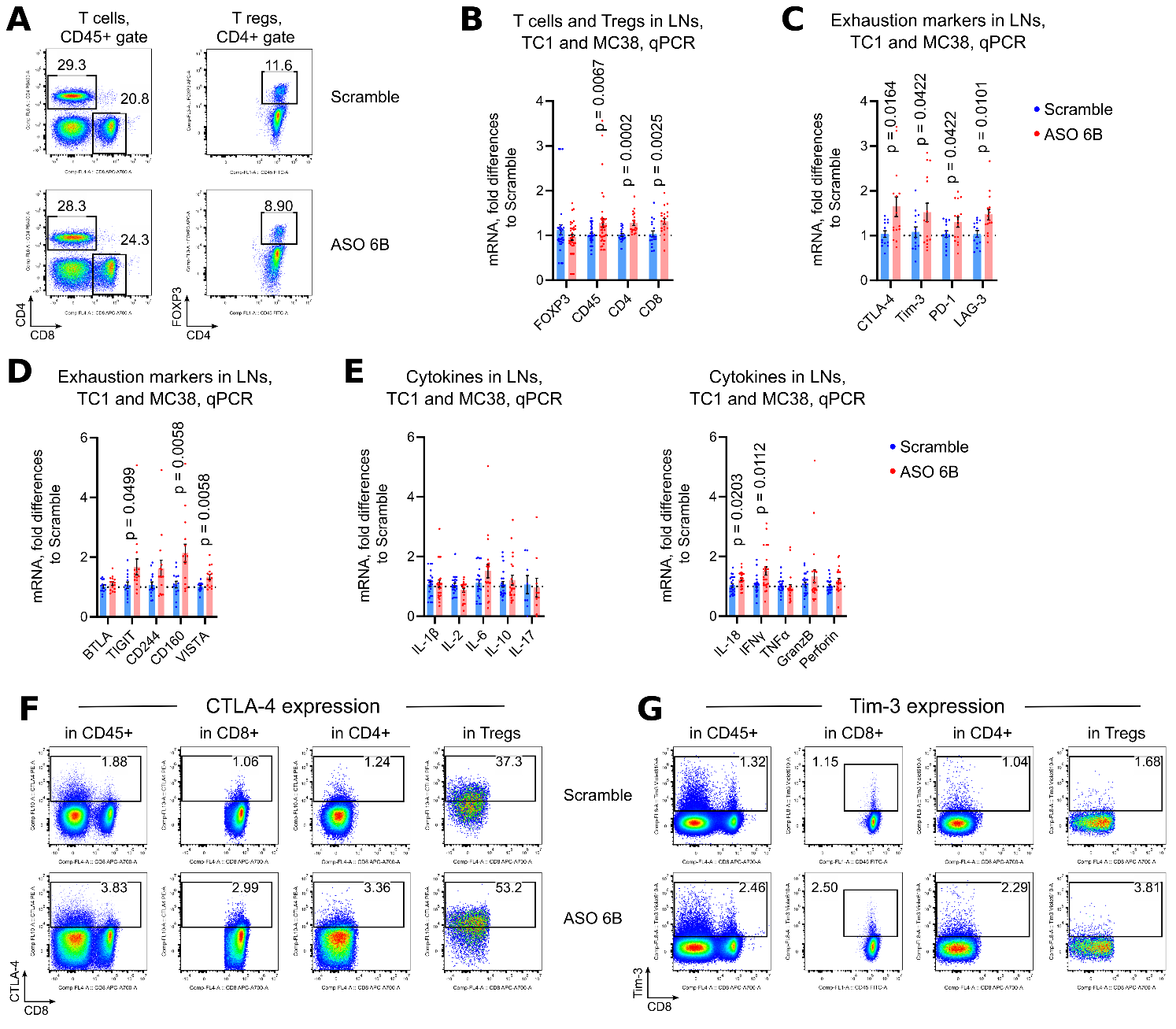
Figure S8. A, F-G**, 28 LNs from TC1 tumors mice and 28 LNs from MC38 mice as in Figure 5 B-C, E-G, were evaluated by flow cytometry. **A**, representative plots of T cells (left) and Treg (right) staining in MC38 LNs. **B-E**, 19 LNs from TC1 tumors mice and 10 LNs from MC38 mice as in Figure 5 A, D, were evaluated by qPCR. **F-G**, representative plots of **F**, CTLA-4 and **G**, Tim-3 expression in LNs.

**B-E**, multiple T-tests FDR Benjamini, Krieger and Yekutieli, with Q = 5%, only results with p<0.05 are shown.


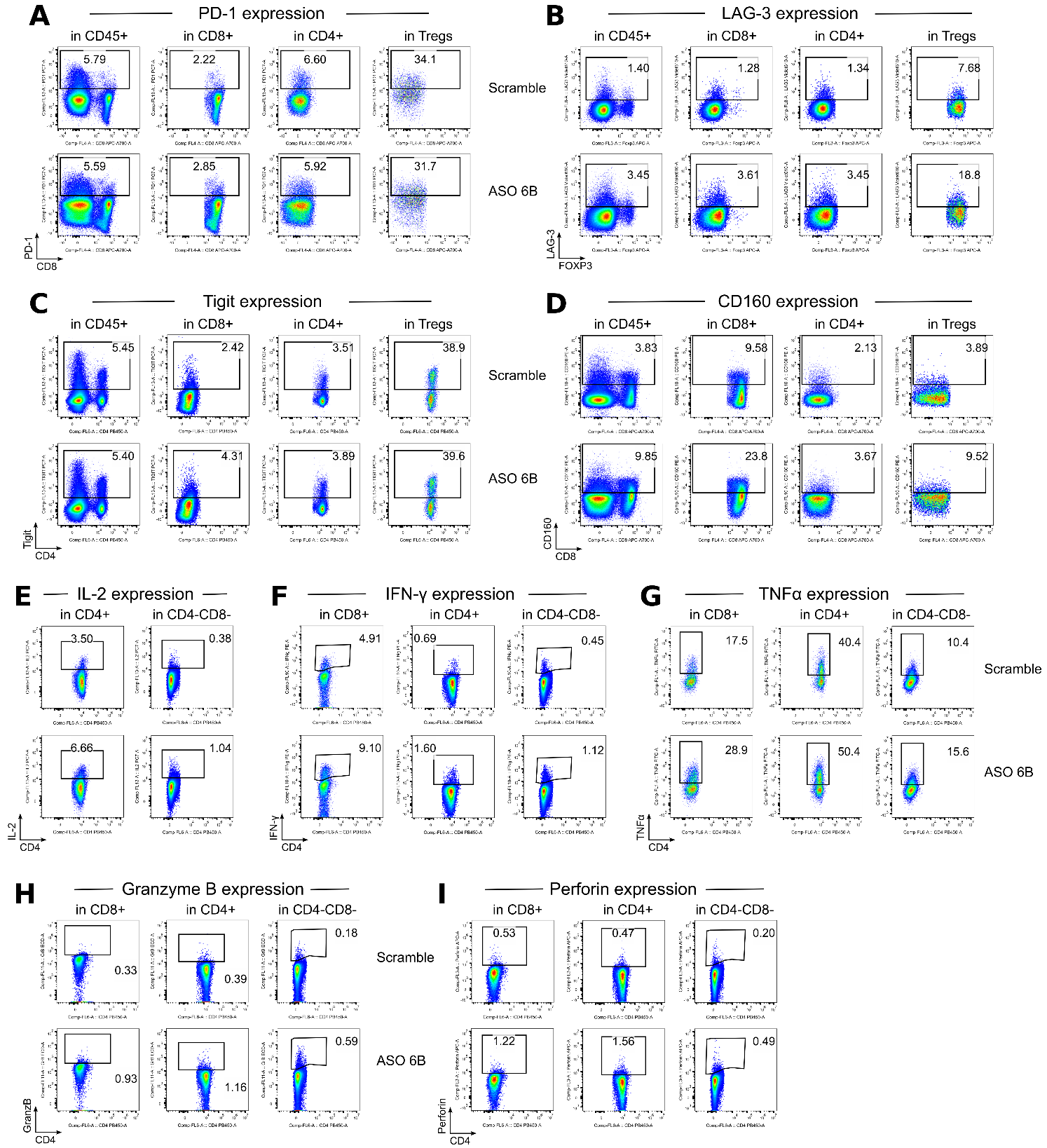
**Figure S9. A-I,** 28 LNs from TC1 tumors mice and 28 LNs from MC38 mice as in Figure 5 B-C, E-G, were evaluated by flow cytometry. Representative flow cytometry plots for **A**, PD-1;  **B**, LAG-; **C**, Tigit and **D**, CD160 expression in LNs are shown. **E-I**, LNs cells were stimulated for 4 h with PMA/ionomycin with Monensin. Representative flow cytometry plots for **E**, IL-2; **F**, IFNγ; **G**, TNFα; **H**, Granzyme B and **I**, Perforin production in LNs are shown.

**
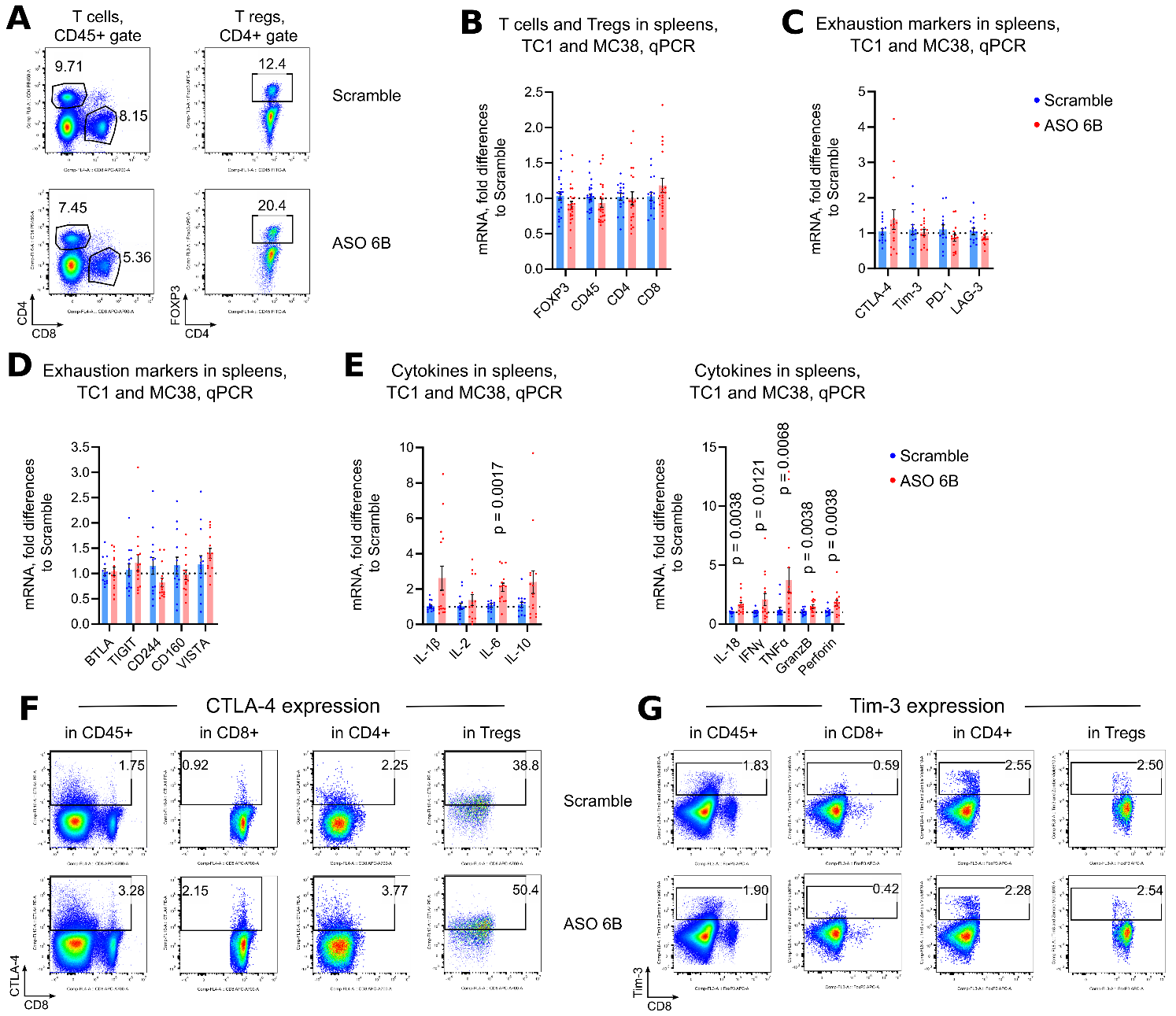
**

**Figure S10. A, F-G**, 28 spleens from TC1 tumors mice and 28 spleens from MC38 mice as in Figure 5 B-C, E-G, were evaluated by flow cytometry. **A**, representative plots of T cells (left) and Treg (right) staining in MC38 spleens. **B-E**, 19 spleens from TC1 tumors mice and 10 spleens from MC38 mice as in Figure 5 A, D, were evaluated by qPCR. **F-G**, representative plots of **F**, CTLA-4 and **G**, Tim-3 expression in the spleens.

**B-E**, multiple T-tests FDR Benjamini, Krieger and Yekutieli, with Q = 5%, only results with p<0.05 are shown.


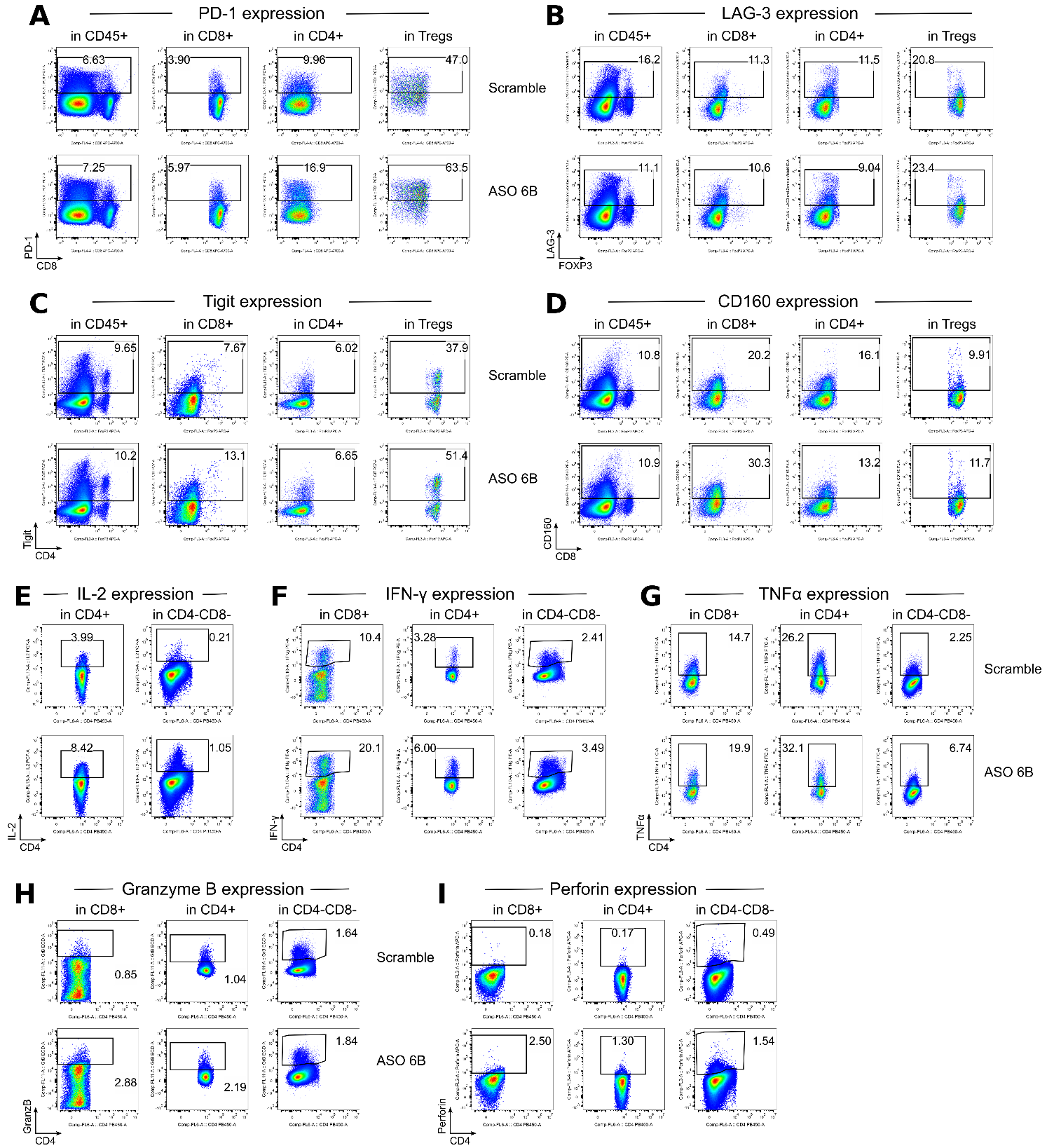
**Figure S11. A-I,** 28 spleens from TC1 tumors mice and 28 spleens from MC38 mice as in Figure 5 B-C, E-G, were evaluated by flow cytometry. Representative flow cytometry plots for **A**, PD-1; **B**, LAG- 3; **C**, Tigit and **D**, CD160 expression in the spleens are shown. **E-I**, splenocytes were stimulated for 4 h with PMA/ionomycin with Monensin. Representative flow cytometry plots for **E**, IL-2; **F**, IFNγ; **G**, TNFα; **H**, Granzyme B and **I**, Perforin production in the spleens are shown.

**
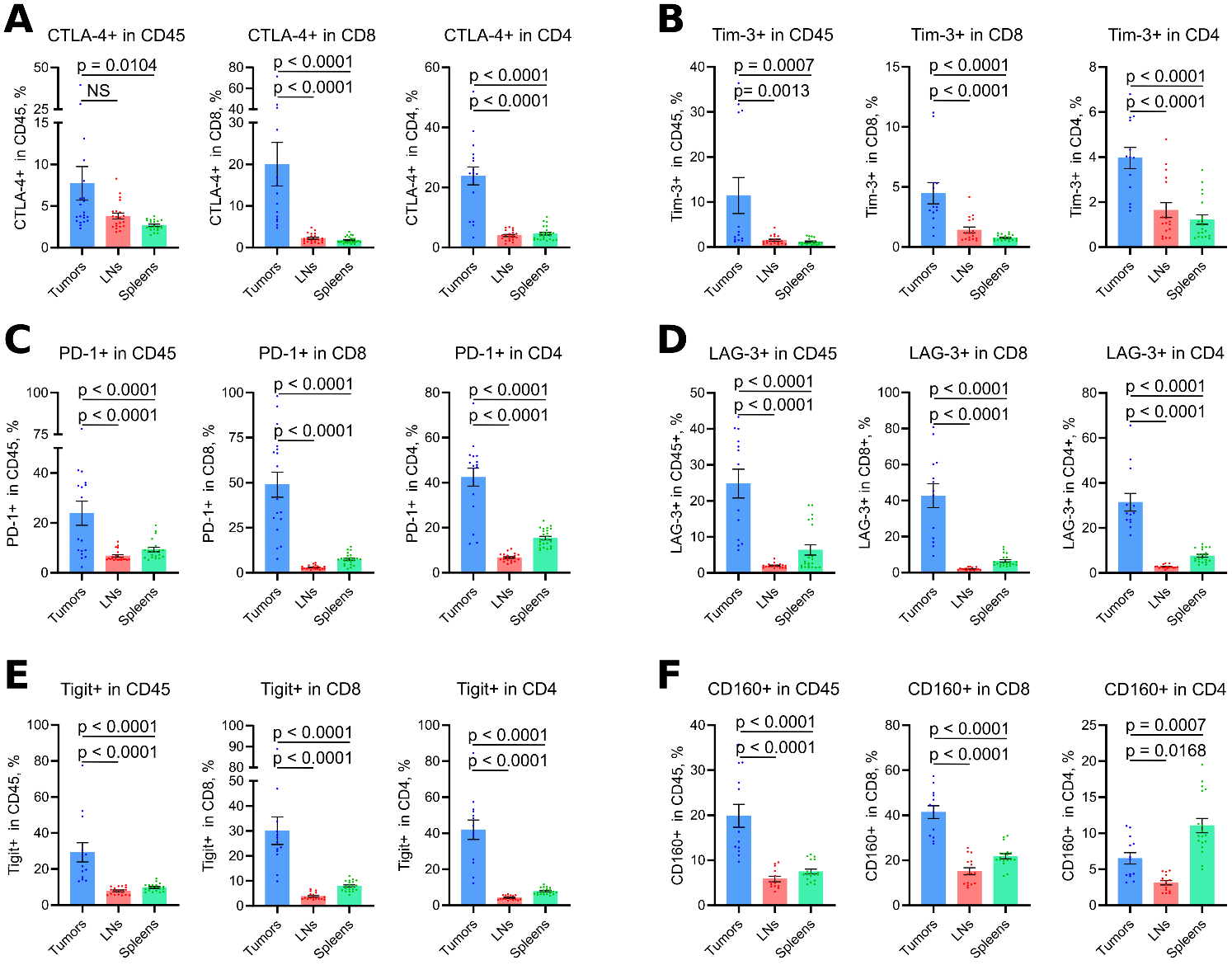
**

**Figure S12. A-F**, flow cytometry data of scramble treated TC1 and MC38 tumor bearing mice as in Figure 5 B-C were compared. Expression levels of exhaustion markers in the tumors, spleens and LNs of TC1 and MC38 tumor bearing mice with corresponding statistics are shown (no Z scoring).

**A-F**, ordinary one-way ANOVA with Tukey’s multiple comparison test.

**
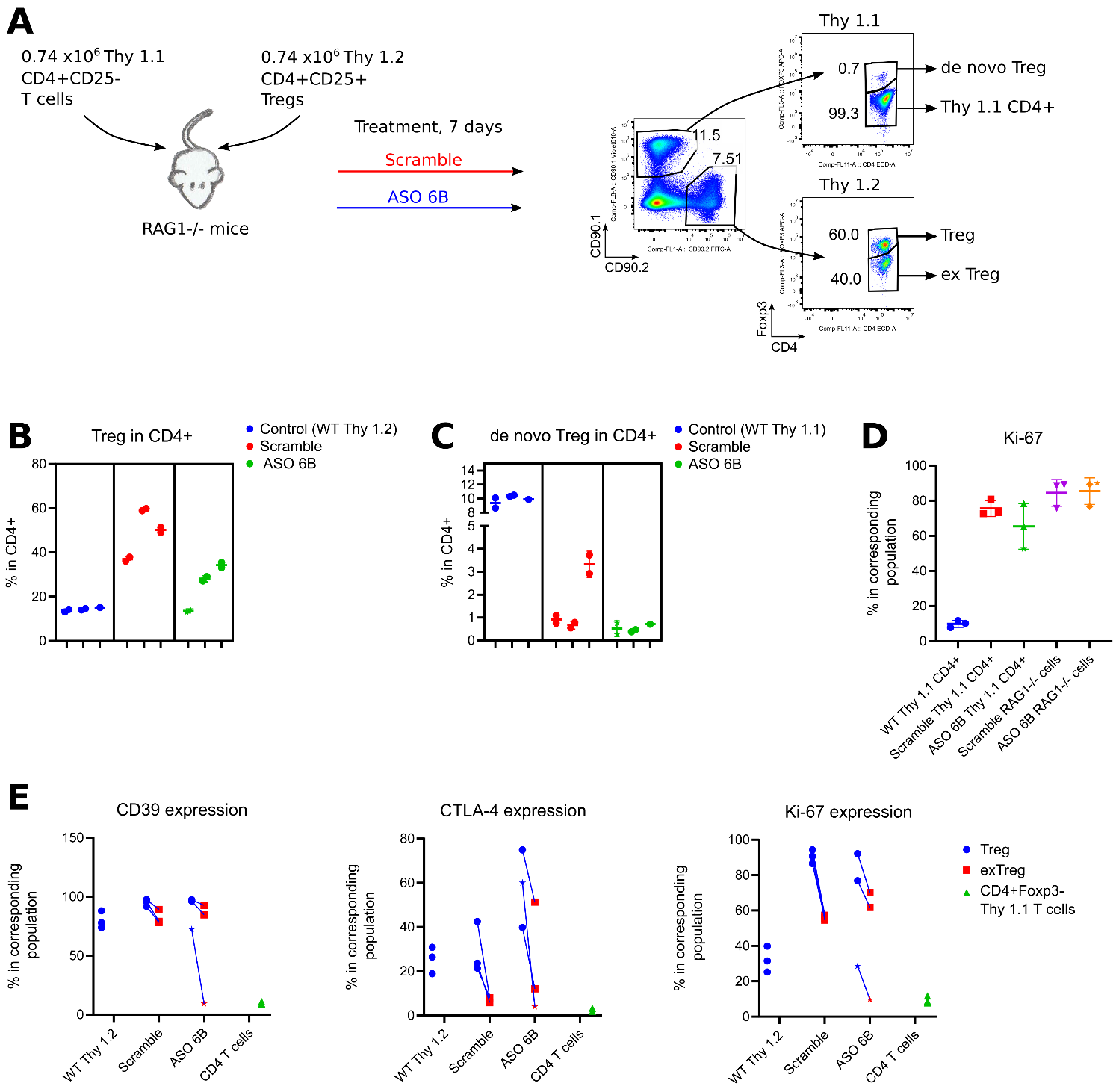
Figure S13. A-E,** Six RAG1-/- mice were injected with a mix of 0.74x10^6^ Thy1.2 Tregs and 0.74x10^6^ Thy1.1 CD4+CD25- conventional T cells, then treated i.p. with Scramble or ASO 6B 50 mg/kg daily for 7 days. At day 8^th^, spleens were harvested and evaluated by flow cytometry. **A**, design of the experiment and a representative flow cytometry plot, showing four resulting CD4+ populations. In Thy1.1+ cells, CD4+FOXP3+ subset represents “de novo” Tregs, i.e. T cells that were FOXP3 negative and upregulated FOXP3 expression in vivo. CD4+FOXP3- subset represents the conventional CD4+ T cells. In Thy1.2+ cells, CD4+FOXP3+ subset represents Tregs, and CD4+FOXP3- subset represents ex Tregs. In ASO 6B treated group, we had to sacrifice one mouse at day 6^th^ of treatment, it has the signs of distress with visible liver inflammation and pancreatitis, probably related with failure in i.p. injection. Data from that mouse shown as stars in all graphs. **B**, expression of FOXP3 in Thy1.2 CD4+ cells in mice that were used for Treg isolation (called “Control WT Thy1.2”), and percent of FOXP3+ Tregs in Thy1.2 CD4+ cells in the end of treatment by Scramble or ASO 6B. There is an apparent trend for decrease Treg numbers in ASO 6B treated mice, although it was not significant by nested T test (p = 0.0585). **C**, expression of FOXP3 in Thy1.1 CD4+ cells in mice that were used for CD4+CD25- T cells isolation (called “Control WT Thy1.1”) and expression of FOXP3 in Thy1.1 CD4+ cells, i.e. percent of “de novo” Tregs. There is no apparent differences in the exception of one Scramble mouse. B,C – data combined from two flow cytometry panels. **D**, rate of in vivo divisions, evaluated by Ki-67 expression in corresponding populations of cells. “WT Thy1.1 CD4+” are cells in mice that were used for CD4+CD25- T cells isolation prior to any manipulations. **E**, comparison of CD39, CTLA-4 and Ki-67 expression in two subsets of Tregs, stable Thy1.2 CD4+FOXP3+ cells (“Treg”) and Thy1.2 CD4+ cells which lost FOXP3 expression (“ex Treg”). Data within each individual mouse are connected with a line. For comparison, the expression of the same markers are shown for non-manipulated Thy1.2 Tregs from non-treated mice (“WT Thy 1.2”) and for non-manipulated Thy1.1 CD4+FOXP3- cells from non-treated mice (“CD4 T cells”).


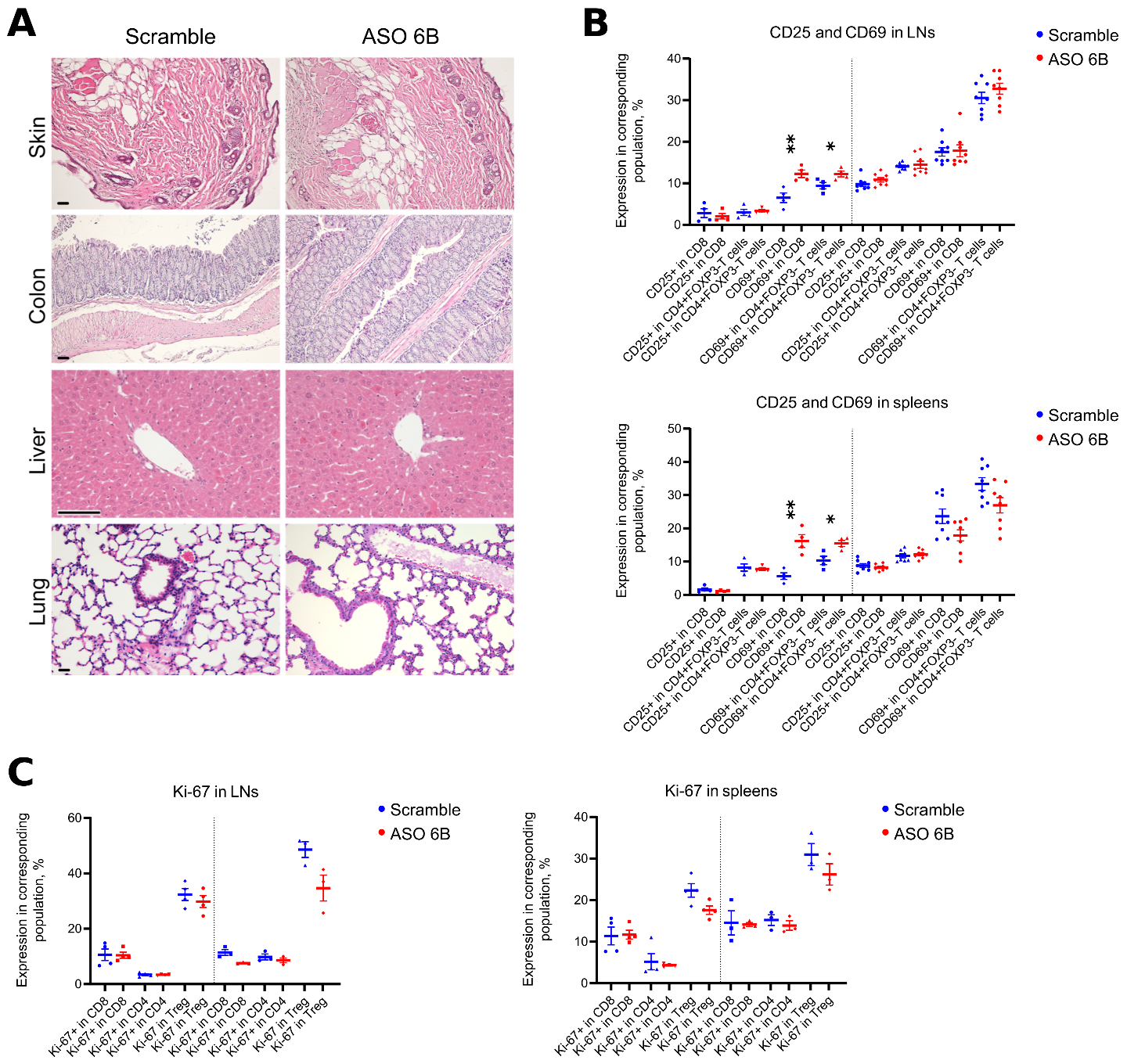


**Figure S14**. **A**, Normal histologic appearance of tissues in TC1 tumor bearing mice treated with Scramble or ASO 6B for 21 days; H&E-stained sections of skin, colon, liver and lung, representative of 10 mice/group are shown (scale bar = 100 µ). **B-C**, evaluation of LNs and spleens in TC1 mice at day 14^th^ after tumor inoculation (on the left at each graph) and in MC38 mice at day 18^th^ after tumor inoculation (on the right at each graph). **B**, CD25 and CD69 and **C**, Ki-67 expression in corresponding populations.

**B, C**, except for the Ki-67 in the spleens – T-tests. **B**, Ki-67 in the spleens – Mann-Whitney tests. * p<0.05, ** p<0.01

# Supplementary Tables

**Table S1. Antibodies and reagents**

| **Flow cytometry** | | | | | | | |
| --- | --- | --- | --- | --- | --- | --- | --- |
| **#** | **Name** | **Clone/catalog #** | | **Color/Type/PRID** | | | **Manufacturer** |
| **Mouse and human** | | | | | | | |
| 1 | CFSE | cat# C1157 | | 492/517 nm | | | Life Technologies |
| 2 | Live/dead fixable | cat# L34957 | | 405/525 nm | | | Life Technologies |
| 3 | Zombie Yellow fixable | cat# 423103 | | 405/572 nm | | | Biolegend |
| 4 | Zombie Aqua fixable | cat#423102 | | 405/516 | | | Biolegend |
| 5 | Ghost Dye, violet 510 | cat#13-0870-T100 | | 405/510 | | | Tonbo Bioscience |
| 6 | Monocytes blocking | cat#426102 | | True-Stain Monocyte Blocker | | | Biolegend |
| 7 | Cytokines assay | cat#420701 | | Monensin Solution | | | Biolegend |
| 8 | Fix/perm | cat#562574 | | Transcription Factor Buffer Set (for FOXP3, CTLA-4, Ki-67) | | | BD Biosciences |
| 9 | Fix/perm | cat# 554714 | | BD Cytofix/Cytoperm™ Fixation/Permeabilization Solution kit (for cytokines) | | | BD Biosciences |
| 10 | Ki-67 | cat#561284 | | PerCP-Cy5.5 (RRID:AB_10611574) | | | BD Biosciences |
| **Human** | | | | | | | |
| 11 | CD4 | RPA-T4 | | PE-CF594 | | | BD Biosciences |
| 12 | CD4 | SK3 | | APC (RRID:AB_2028488), FITC (RRID:AB_2616621), Pacific Blue | | | Biolegend |
| 13 | CD4 | OKT4 | | eFluor 450 | | | eBioscience |
| 14 | CD8 | HIT8a | | PerCP/Cy5.5, FITC | | | Biolegend |
| 15 | CD8a | RPA-T8 | | PE-CF594 (RRID:AB_2869914) | | | BD Biosciences |
| 16 | CD25 | M-A251 | | APC | | | Biolegend |
| 17 | CD39 | eBioA1 (A1) | | Pe-Cy7 (RRID:AB_1582280) | | | eBioscience |
| 18 | CD45 | HI30 | | FITC (RRID:AB_314394), Brilliant Violet 605™ | | | Biolegend |
| 19 | CD152 (CTLA-4) | BNI3 | | PE (RRID:AB_396628) | | | BD Biosciences |
| 20 | CD160 | BY55 | | PE | | | Biolegend |
| 21 | CD223 (LAG-3) | 7H2C65 | | APC | | | Biolegend |
| 22 | CD272 (BTLA) | J168-540 | | BV421 | | | BD Biosciences |
| 23 | CD279 (PD-1) | EH12.2H7 | | Alexa Fluor® 700 | | | Biolegend |
| 24 | FOXP3 | PCH101 | | eFluor 450 (RRID:AB_1834364), PE, Pe-Cy7 | | | eBioscience |
| 25 | GARP | 7B11 | | PE (RRID:AB_10900640) | | | Biolegend |
| 26 | Helios | 22F6 | | APC (RRID:AB_10662900), FITC (RRID: AB_10662745), PE (RRID:AB_10660749), | | | Biolegend |
| 27 | TIGIT | MBSA43 | | PE | | | eBioscience |
| 28 | TIM-3 | 344823 | | APC | | | R&D systems |
| 29 | Fc blocking | cat#422302 | | Human TruStain FcX™ (RRID:AB_2818986) | | | Biolegend |
| **Mouse** | | | | | | | |
| 30 | CD4 | RM4-5 | | Pacific Blue | | | Biolegend |
| 31 | CD4 | RM4-5 | | PE-CF594 | | | BD Biosciences |
| 32 | CD8 | 53-6.7 | | Alexa Fluor® 700, Brilliant Violet 605, FITC | | | Biolegend |
| 33 | CD25 | PC61 | | Alexa Fluor® 700, Pe-Cy7 | | | Biolegend |
| 34 | CD45 | 30-F11 | | Alexa Fluor® 700, FITC, Pacific Blue | | | Biolegend |
| 35 | CD69 | H1.2F3 | | APC, PE-CF594 (RRID:AB_11154217) | | | Biolegend, BD Bioscience |
| 36 | CD90.1 | OX-7 | | Brilliant Violet 605 (RRID:AB_2562644) | | | Biolegend |
| 37 | CD90.2 | 30-H12 | | Alexa Fluor® 488 (RRID:AB_492886) | | | Biolegend |
| 38 | CD152 (CTLA-4) | UC10-4F10 | | PE | | | BD Biosciences |
| 39 | CD160 | 7H1 | | PE | | | Biolegend |
| 40 | CD223 (LAG-3) | eBioC9B7W | | Super Bright™ 600 | | | eBioscience |
| 41 | CD279 (PD-1) | 29F.1A12 | | Pe-Cy7 | | | Biolegend |
| 42 | Foxp3 | FJK-16s | | APC | | | eBioscience |
| 43 | Granzyme B | GB11 | | PE-CF594 | | | BD Biosciences |
| 44 | IFN-γ | XMG1.2 | | PE | | | Biolegend |
| 45 | IL-2 | JES6-5H4 | | Pe-Cy7 | | | Biolegend |
| 46 | Perforin | S16009B | | APC | | | Biolegend |
| 47 | Tigit | GIGD7 | | Pe-Cy7 | | | eBioscience |
| 48 | Tim-3 | 8B.2C12 | | Super Bright™ 600 | | | eBioscience |
| 49 | TNF-α | MP6-XT22 | | Alexa Fluor® 488 | | | Biolegend |
| 50 | Fc blocking | Purified | | Anti-mouse CD16/32 | | | Biolegend |
| **Stimulation** | | | | | | | |
| 51 | CD3 mAbs-coated beads | OKT3  and  Dynabeads | | MACS GMP pure, human (RRID:AB_2904535) and M-450 Tosylactivated beads | | | Miltenyi Biotec and  Life Technologies |
| 52 | CD3/28 beads | 11131D | | Dynabeads™ Human T-Activator CD3/CD28 | | | Gibco |
| 53 | PMA | cat# P8139-1MG | | Phorbol 12-myristate 13-acetate | | | Sigma-Aldrich Co |
| 54 | Ionomycin | cat# I0634-1MG | | Ionomycin calcium salt | | | Sigma-Aldrich Co |
| **RT-qPCR reagents** | | | | | | | |
| **Human and humanized mice** | | | | | **Mouse** | | |
| **#** | **Name** | | **Catalog #** | |  | **Name** | **Catalog #** |
| 55 | 18S (housekeeping) | | 4319413E | | 88 | TBP (TATA-box binding Protein, housekeeping) | Mm00446973_m1 |
| 56 | TBP (TATA-box binding Protein, housekeeping) | | 4333769F | | 89 | Hprt (hypoxanthine guanine phosphoribosyl transferase, housekeeping) | Mm00446968_m1 |
| 57 | SDHA (succinate dehydrogenase complex flavoprotein subunit A, housekeeping) | | Hs00417200_m1 | | 90 | Foxp3 | Mm00475162_m1 |
| 58 | GAPDH | | 4326317E | | 91 | BTLA | Mm00616981_m1 |
| 59 | FOXP3 | | Hs01085834_m1 | | 92 | CD160 | Mm00444461_m1 |
| 60 | BTLA | | Hs00699198_m1 | | 93 | CD244 | Mm00479575_m1 |
| 61 | CD160 | | Hs00199894_m1 | | 94 | CD4 | Mm00442754_m1 |
| 62 | CD244 | | Hs00175569_m1 | | 95 | CD8 | Mm01182107_g1 |
| 63 | CD4 | | Hs01058407_m1 | | 96 | CD45 | Mm01293577_m1 |
| 64 | CD8 | | Hs00233520_m1 | | 97 | CTLA-4 | Mm00486849_m1 |
| 65 | CD45 | | Hs04189704_m1 | | 98 | Granzyme B | Mm00442837_m1 |
| 66 | CTLA-4 | | Hs00175480_m1 | | 99 | Interferon gamma, IFN-γ | Mm01168134_m1 |
| 67 | FOXP1 | | Hs00908900_m1 | | 100 | IL-1β, interleukin 1, beta | Mm00434228_m1 |
| 68 | FOXP2 | | Hs00362818_m1 | | 101 | IL-2, interleukin 2 | Mm00434256_m1 |
| 69 | FOXP4 | | Hs01055269_m1 | | 102 | IL-6, interleukin 6 | Mm00446190_m1 |
| 70 | GARP | | Hs00194136_m1 | | 103 | IL-10, interleukin 10 | Mm01288386_m1 |
| 71 | Granzyme B | | Hs00188051_m1 | | 104 | IL-17a, interleukin 17a | Mm00439618_m1 |
| 72 | Interferon gamma, IFN-γ | | Hs00989291_m1 | | 105 | IL-18, interleukin 18 | Mm00434225_m1 |
| 73 | IL-1β, interleukin 1, beta | | Hs00174097_m1 | | 106 | LAG-3 | Mm00493071_m1 |
| 74 | IL-2, interleukin 2 | | Hs00174114_m1 | | 107 | PD-1, PDCD1 | Mm01285676_m1 |
| 75 | IL-6, interleukin 6 | | Hs00174131_m1 | | 108 | Perforin PRF1 | Mm00812512_m1 |
| 76 | IL-7, interleukin 7 | | Hs00174202_m1 | | 109 | TIGIT | Mm03807522_m1 |
| 77 | IL-10, interleukin 10 | | Hs00961622_m1 | | 110 | Tim-3 | Mm00454540_m1 |
| 78 | IL-17a, interleukin 17a | | Hs99999082_m1 | | 111 | TNFα | Mm00443258_m1 |
| 79 | IL-18, interleukin 18 | | Hs01038788_m1 | | 112 | VISTA | Mm00472312_m1 |
| 80 | LAG-3 | | Hs00958444_g1 | |  |  |  |
| 81 | PD-1, PDCD1 | | Hs01550088_m1 | |  |  |  |
| 82 | Perforin PRF1 | | Hs00169473_m1 | |  |  |  |
| 83 | TGFβ (transforming growth factor, beta 1) | | Hs00998133_m1 | |  |  |  |
| 84 | TIGIT | | Hs00545087_m1 | |  |  |  |
| 85 | Tim-3 | | Hs00262170_m1 | |  |  |  |
| 86 | TNFα | | Hs01113624_g1 | |  |  |  |
| 87 | VISTA | | Hs00735289_m1 | |  |  |  |

**Table S2. Statistics of PCA analysis, TC1 and MC38 tumors, flow cytometry data**

| **KMO and Bartlett's Test** | | | | | | | |  |  |  |
| --- | --- | --- | --- | --- | --- | --- | --- | --- | --- | --- |
| Kaiser-Meyer-Olkin Measure of Sampling Adequacy. | | | | | | .786 | |  |  |  |
| Bartlett's Test of Sphericity | | | Approx. Chi-Square | | | 96.145 | |  |  |  |
|  |  |  | df | | | 36 | |  |  |  |
|  |  |  | Sig. | | | .000 | |  |  |  |
| **Total Variance Explained** | | | | | | | | | | |
| Component | Initial Eigenvalues | | | | Extraction Sums of Squared Loadings | | | | | Rotation Sums of Squared Loadings |
|  | Total | % of Variance | | Cumulative % | Total | | % of Variance | | Cumulative % | Total |
| 1 | 4.728 | 52.532 | | 52.532 | 4.728 | | 52.532 | | 52.532 | 4.494 |
| 2 | 1.489 | 16.546 | | 69.077 | 1.489 | | 16.546 | | 69.077 | 2.155 |
| 3 | .891 | 9.896 | | 78.973 |  | |  | |  |  |
| 4 | .798 | 8.867 | | 87.839 |  | |  | |  |  |
| 5 | .434 | 4.822 | | 92.661 |  | |  | |  |  |
| 6 | .264 | 2.931 | | 95.592 |  | |  | |  |  |
| 7 | .180 | 2.002 | | 97.594 |  | |  | |  |  |
| 8 | .136 | 1.514 | | 99.109 |  | |  | |  |  |
| 9 | .080 | .891 | | 100.000 |  | |  | |  |  |

| **Pattern Matrix, Direct Oblimin rotation** | | |
| --- | --- | --- |
|  | Component | |
|  | 1 | 2 |
| Treg in CD4+ | .577 | -.416 |
| LAG-3+ in CD45+ | .721 | -.325 |
| LAG-3+ in CD4 | .943 |  |
| LAG-3+ in Treg | .934 |  |
| CTLA-4+ in CD4 | .672 | -.446 |
| Tim-3+ in CD4 | .720 |  |
| PD-1+ in CD4+ | .361 | -.742 |
| PD-1+ in CD8+ | .688 |  |
| IFNg+ in CD4-CD8- |  | .784 |

**4 Supplementary References**

1. Zhang X, Castanotto D, Liu X, Shemi A, Stein CA. Ammonium and arsenic trioxide are potent facilitators of oligonucleotide function when delivered by gymnosis. Nucleic Acids Res. 2018;46(7):3612-24.
